# Expanded base editing in rice and wheat using a Cas9-adenosine deaminase fusion

**DOI:** 10.1186/s13059-018-1443-z

**Published:** 2018-05-29

**Authors:** Chao Li, Yuan Zong, Yanpeng Wang, Shuai Jin, Dingbo Zhang, Qianna Song, Rui Zhang, Caixia Gao

**Affiliations:** 1 State Key Laboratory of Plant Cell and Chromosome Engineering, Center for Genome Editing, Institute of Genetics and Developmental Biology, Chinese Academy of Sciences, Beijing, China; 2 University of Chinese Academy of Sciences, Beijing, China

**Keywords:** Cas9-adenosine deaminase, sgRNA forms, Rice, Wheat, Herbicide resistance

## Abstract

Nucleotide base editors in plants have been limited to conversion of cytosine to thymine. Here, we describe a new plant adenine base editor based on an evolved tRNA adenosine deaminase fused to the nickase CRISPR/Cas9, enabling A•T to G•C conversion at frequencies up to 7.5% in protoplasts and 59.1% in regenerated rice and wheat plants. An endogenous gene is also successfully modified through introducing a gain-of-function point mutation to directly produce an herbicide-tolerant rice plant. With this new adenine base editing system, it is now possible to precisely edit all base pairs, thus expanding the toolset for precise editing in plants.

**Electronic supplementary material:**

The online version of this article (10.1186/s13059-018-1443-z) contains supplementary material, which is available to authorized users.

## Background

The CRISPR (clustered regularly interspaced short palindromic repeat) system has been used to edit a variety of plant species [1]. CRISPR/Cas9 and CRISPR/Cpf1 typically produce double strand breaks (DSBs) that result in mutant plants with either gene knock-outs (via non-homologous end joining (NHEJ)) or gene replacements and insertions (via homology-directed repair (HDR)) [2, 3]. Base editing is a unique genome editing system that creates precise and highly predictable nucleotide substitutions at genomic targets without requiring DSBs, or donor DNA templates, or depending on NHEJ and HDR [4]. Base editing is more efficient than HDR-mediated base pair substitution, and produces fewer undesirable mutations in the target locus [5]. The most commonly used base editing systems, such as BE3 [6], BE4 [7], Targeted-AID [8], and dCpf1-BE [9], use Cas9 or Cpf1 variants to recruit cytidine deaminases that exploit DNA mismatch repair pathways and generate specific C to T substitutions. This base-editing technology has already been used in a wide variety of cell lines and organisms [4, 5]. Recently, adenine base editors (ABE), developed by fusing an evolved tRNA adenosine deaminase with SpCas9 nickase (D10A), were shown to generate A•T to G•C conversions when directed by single guide RNAs (sgRNAs) to genomic targets in human cells [10].

In this report, we adapted this method and optimized an ABE for application in plant systems, demonstrating its high efficiency in creating targeted point mutations at multiple endogenous loci in rice and wheat.

## Results

**Fig. 1:**
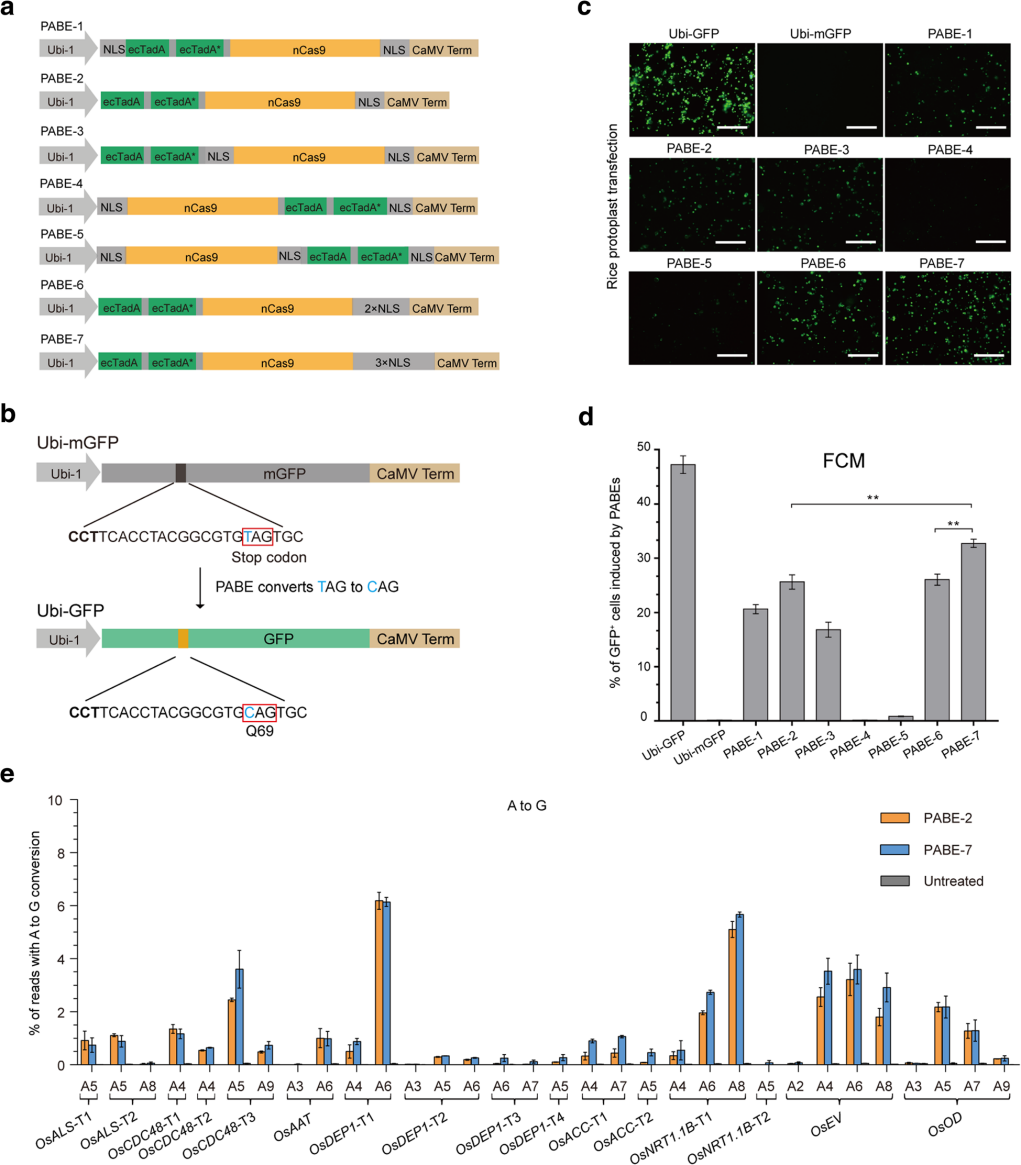
Comparison of A•T to G•C base-editing efficiency in rice protoplasts using seven PABE constructs. **a** The seven plant adenine base editing (PABE) constructs. **b** Diagram of the GFP reporter system for comparing the activities of the seven PABE constructs in rice protoplasts. The TAG stop codon (whose conversion to CAG restores GFP protein production) and CAG triplets are shown in the *red box*. **c** Plant ABE-induced conversion of mGFP to GFP in rice protoplasts by the seven PABE constructs. Seven fields of protoplasts transformed with the relevant PABE construct, sgRNA-mGFP and Ubi-mGFP vectors. Ubi-GFP and Ubi-mGFP served as controls. *Scale bars*, 150 μm. **d** The frequencies (percentage) of A to G conversion in the target region of the mGFP coding sequence were measured by flow cytometry (FCM) on three independent biological replicates (*n* = 3). All values represent means ± standard error of the mean (s.e.m.). **P < 0.01. **e** Frequencies of targeted single A to G conversion in reads of the 16 target sites by PABE-2 and PABE-7 in rice protoplasts. An untreated protoplast sample was used as control. Each frequency (mean ± s.e.m.) was calculated using the data from three independent biological replicates (*n* = 3)

We used ABE7.10, a fusion of an adenosine deaminase (ecTadA-ecTadA*) with nCas9 (D10A), which base edits A•T to G•C accurately in human cells [10]. To develop an efficient ABE for plant cells, we constructed seven ABE fusion proteins. The seven proteins, named PABE-1 to PABE-7, varied in the position of the adenosine deaminase and the number and locations of nuclear localization sequences (NLSs; Fig. 1a; Additional file 1: Sequences). All the PABE constructs were codon-optimized for cereal plants, and placed under control of the maize *Ubiquitin-1* promoter (Ubi-1).

Editing efficiencies of the PABE constructs were first tested using a green fluorescent protein (GFP) reporter that contained a mutation within the expression cassette converting the Gln-69 codon (CAG) for GFP into a stop codon (TAG) (Fig. 1b). This mutated gene, termed *mGFP*, produces active GFP when the stop codon is corrected by a T to C single nucleotide substitution (TAG to CAG), thus allowing mutagenesis efficiency to be measured as the frequency of GFP-expressing cells (Fig. 1b). We designed an sgRNA-mGFP with the desired T at position 6 (T_6_) of the protospacer, counting from the distal end to the protospacer-adjacent motif (PAM), based on the ABE7.10 deamination window in human cells [10] (Fig. 1b; Additional file 2: Table S1). Each PABE construct was co-transfected with sgRNA-mGFP and Ubi-mGFP into rice protoplasts by PEG-mediated transformation [11].

At 24 h post-transfection, GFP fluorescence was reliably detected in cells treated with the following five of the seven test constructs: PABE-1, PABE-2, PABE-3, PABE-6, and PABE-7 (Fig. 1c). Flow cytometry (FCM) analyses showed that the percentages of fluorescent cells ranged from 0.1 to 32.8% (Fig. 1d). Three copies of the NLS at the C-terminus of nCas9 (PABE-7) gave the highest yield of GFP-expressing cells, higher than PABE-2, which was similar to the construct used in human cells [10] and the other PABE constructs (Fig. 1c, d). These results also showed that putting ecTadA-ecTadA* adenosine deaminase at the C-terminus of nCas9 (PABE-4 and PABE-5) renders the plant ABE system ineffective (Fig. 1c, d).

To further compare the editing efficiency of PABE-2 and PABE-7, we targeted 16 rice endogenous genomic sites (Fig. 1e; Additional file 2: Table S1). A to G base editing of the respective genes in protoplasts was assessed by next-generation sequencing (100,000–220,000 reads per locus). PABE-7 was identified to offer modestly higher base editing efficiency, about 1.1-fold average increase in A•T to G•C conversion at each site over PABE-2 (Fig. 1e; Additional file 2: Table S3). Taken together, these results demonstrate that the plant ABE system can induce A to G conversions in rice, and that the presence of three NLS at the C-terminus of nCas9 maximizes editing efficiency.

**Fig. 2:**
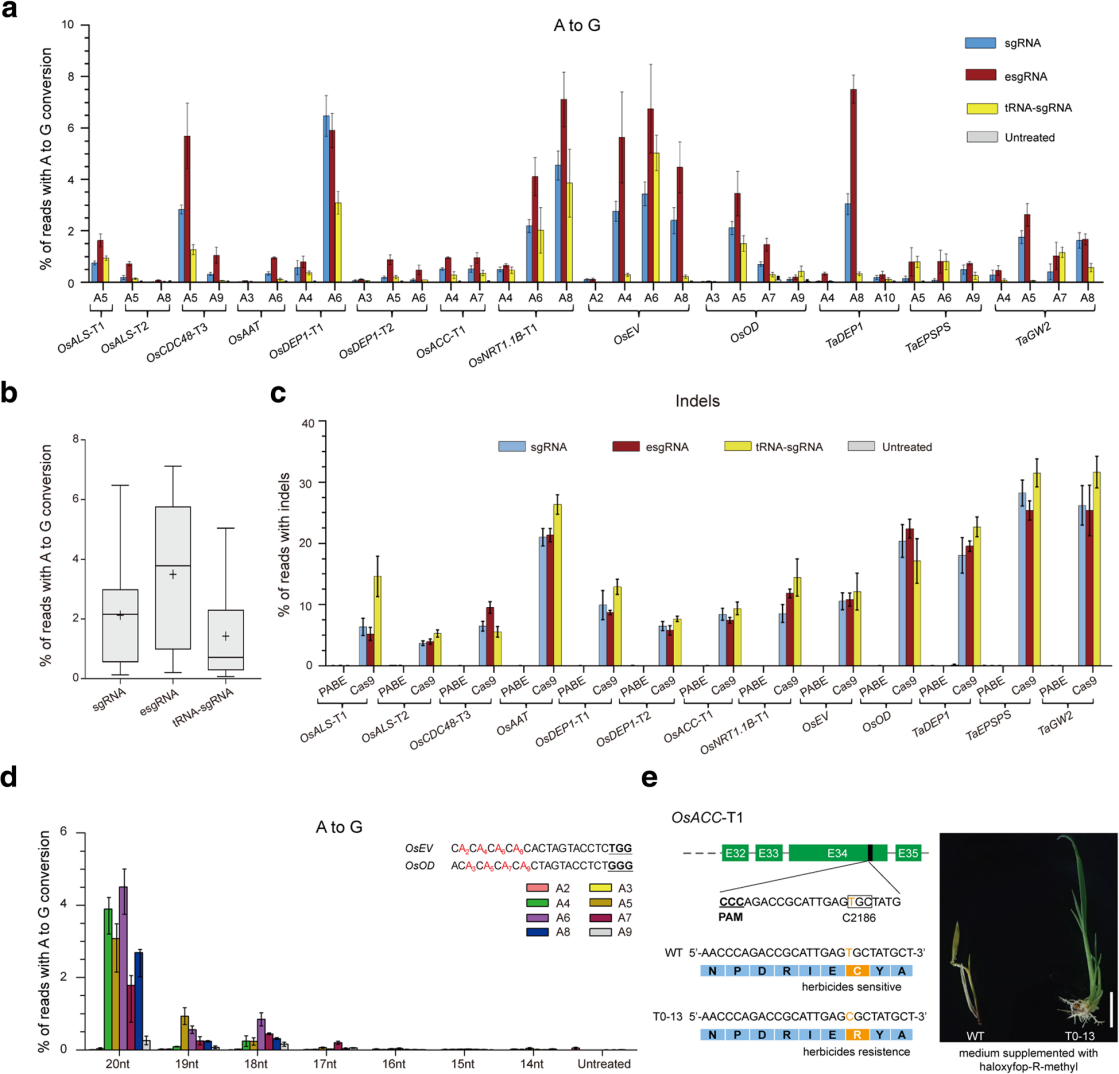
Analysis of PABE-7 activity on endogenous genes using different sgRNA expression constructs. **a** Frequencies of targeted single A to G conversions in the 13 target sites of rice and wheat genes. The native forms of sgRNA, esgRNA, and tRNA-sgRNA were used. **b** Summary of the A to G conversion activities of PABE-7 in **a**. **c** Frequencies of indels in the 13 target sites of rice and wheat genes. **d** The effect of spacer length of sgRNA on editing efficiency. A to G editing frequencies induced by the PABE-7 and esgRNAs of different length varying from 14 to 20 nucleotides were determined at protospacer positions 2–9. In **a**, **c,** and **d**, an untreated protoplast sample was used as control and each frequency (mean ± standard error of the mean) was calculated using the data from three independent biological replicates (*n* = 3). **e** *OsACC*-T1 with C2186R substitution confers resistance to herbicide. Sequence alignment comparing WT *OsACC-*T1 with that in the T0–13 mutant. Phenotypes of T0–13 with C2186R substitution in the regeneration medium supplemented with 0.086 ppm haloxyfop-R-methyl. *Scale bars*, 1 cm

To identify the optimal form of sgRNA for PABE-7 activity, various sgRNA modifications were tested over a broad range of endogenous loci. Previous work has shown that modifications to the sgRNA sequence (known as sgRNA^(F + E)^, enhanced sgRNA, or esgRNA) [12] or tRNA-sgRNA expression system [13, 14] can enhance CRISPR/Cas9 genome editing. We therefore compared the base editing activities of the three sgRNA forms (native sgRNA, esgRNA, tRNA-sgRNA) at ten and three endogenous genomic target sites in rice and wheat, respectively (Fig. 2a; Additional file 2: Figure S1 and Table S1). The protospacers targeting these endogenous genes were individually cloned into the three sgRNA structures and co-transformed with PABE-7 into either rice or wheat protoplasts. Wild-type Cas9 (WT Cas9) was used as a control to produce deletion and/or insertion mutations (indels). A to G conversion was observed at all 13 target sites for each combination of PABE-7 and sgRNA expression system, with effective editing frequency spanning positions 4 to 8 within the protospacer (Fig. 2a). Of the three sgRNA constructs, esgRNA showed the highest base editing efficiency in a large majority of the tests ranging from 0.1–7.5% in both rice and wheat (Fig. 2a). The average efficiency of esgRNA for the 13 target sites was about twofold higher than that of the native sgRNA, and threefold higher than that of the tRNA-sgRNA (Fig. 2b), which is consistent with the observation that esgRNA increases the stability and promotes complexing with the Cas9 protein [12]. We observed only A to G conversions, with no evidence of undesired editing at any of the rice and wheat genomic on-target loci (< 0.02%; Additional file 2: Figures S2 and S3), and a much lower frequency of indels (< 0.1%) than with WT Cas9 (3.3–31.6%) (Fig. 2c). To summarize, the PABE-7 base editing construct, together with the esgRNA, induces A to G substitutions efficiently and with high fidelity at multiple loci in rice and wheat.

We also tested the effect of spacer length of the esgRNA on base editing efficiency by targeting *OsEV* and *OsOD*, and found that the esgRNAs with canonical 20-nucleotide spacers showed the highest conversion efficiency (Fig. 2d; Additional file 2: Table S3). At both target sites, esgRNAs with spacer lengths ranging from 14 to 19 nucleotides showed substantially decreased or undetectable A to G base editing activities (< 0.9%) compared with the esgRNAs with canonical 20-nucleotide spacers (< 4.5%) (Fig. 2d). In addition, the WT Cas9 with 14- to 19-nucleotide spacer lengths of esgRNAs also gave much lower frequencies of indels (0.3–12.6%) than with 20-nucleotide esgRNA (10.8–22.4%) at these two sites (Additional file 2: Figure S4). These results suggest that the 20-nucleotide spacer of esgRNA is essential for the plant ABE system with no tolerance for shorter lengths.

To demonstrate the use of PABE-7 to regenerate rice mutant plants, we targeted six rice genomic loci (*OsACC*-T1, *OsALS-*T1, *OsCDC48*-T3, *OsDEP1*-T1, *OsDEP1*-T2, and *OsNRT1.1B-*T1) (Table 1; Additional file 2: Table S1) using *Agrobacterium*-mediated transformation (Additional file 2: Figure S5a). After examination of PABE-7 with esgRNA (pH-PABE-7-esgRNA)-transformed lines, substitutions were identified in all six sites in the T0 seedlings (Table 1; Additional file 2: Figure S5). The A to G substitution efficiencies varied from 15.8 to 59.1%, and we identified one, six, one, and thirteen homozygous mutant lines for *OsACC-T1*, *OsDEP1*-T1, *OsDEP1*-T2, and *OsNRT1.1B-*T1, respectively (Table 1; Additional file 2: Figure S5). Importantly, we noticed that the PABE-7 conversion frequency with esgRNA was on average 1.7-fold higher than that obtained with the native sgRNA constructs at each site in side-by-side experiments (Table 1), consistent with the results observed with protoplasts (Fig. 2a, b). Among all these six target sites, the effective deamination window (4 to 8) was consistent with the protoplast results. In addition, none of the transgenic rice plants contained any indels or undesired edits at the target site (Additional file 2: Figure S5).

**Table 1:** Mutation frequencies induced by PABE-7 in the T0 rice and wheat plants

Species	Target site	sgRNA form	Number of mutant lines/plants^a^	Number of transgenic rice lines or bombarded embryos of wheat	A•T to G•C frequency (%)^b^	Genotype of mutations	Heterozygous/homozygous
Rice	*OsACC*-T1	sgRNA	9	130	6.9	T_4_ > C_4_ (2); T_4_T_7_ > C_4_C_7_ (7)	9/0
		esgRNA	33	160	20.6	T_4_ > C_4_ (10); T_7_ > C_7_ (2); T_4_T_7_ > C_4_C_7_ (21)	32/1
	*OsALS-*T1	sgRNA	16	184	8.7	A_5_ > G_5_ (16)	16/0
		esgRNA	42	196	21.4	A_4_ > G_4_ (1); A_5_ > G_5_ (41)	42/0
	*OsCDC48*-T3	sgRNA	19	210	9.0	A_5_ > G_5_ (19)	19/0
		esgRNA	60	180	33.3	A_5_ > G_5_ (60)	60/0
	*OsDEP1*-T1	sgRNA	101	217	46.5	A_4_ > G_4_ (2); A_6_ > G_6_ (90); A_4_A_6_ > G_4_G_6_ (9)	88/13
		esgRNA	83	211	39.3	A_4_ > G_4_ (4); A_6_ > G_6_ (73); A_4_A_6_ > G_4_G_6_ (6)	77/6
	*OsDEP1*-T2	sgRNA	5	154	3.2	A_6_ > G_6_ (5)	5/0
		esgRNA	34	215	15.8	A_5_ > G_5_ (1); A_6_ > G_6_ (32); A_3_A_6_ > G_3_G_6_ (1)	33/1
	*OsNRT1.1B*-T1	sgRNA	116	303	38.3	A_6_ > G_6_ (8); A_8_ > G_8_ (30); A_4_A_8_ > G_4_G_8_ (3); A_6_A_8_ > G_6_G_8_ (75)	111/5
		esgRNA	149	252	59.1	A_6_ > G_6_ (6); A_8_ > G_8_ (46); A_4_A_8_ > G_4_G_8_ (2); A_6_A_8_ > G_6_G_8_ (95)	136/13
Wheat	*TaDEP1*	esgRNA	5	460	1.1	A_8_ > G_8_ (4, AaBBDD; 1, AABbDD)	5/0
	*TaGW2*	esgRNA	2	480	0.4	A_5_ > G_5_ (2, AABbDD)	2/0

^a^The number of mutant lines for rice and the number of mutant plants for wheat. ^b^ Based on the number of T0 lines (rice) or plants (wheat) carrying the observed mutations over the total number of T0 transgenic rice lines analyzed or bombarded immature embryos of wheat

Herbicide resistance is an important goal in modern crop breeding as it will reduce the time cost for weeding. In turn, this makes a significant contribution to increasing food productivity and reducing soil degradation. Herbicides often target specific enzymes in metabolic pathways, and mutations in an enzyme can be selected that confer herbicide resistance through a substitution in a single amino acid [15]. Acetyl-coenzyme A carboxylase (*ACC*) is a key enzyme in lipid biosynthesis and it has been shown that a T to C replacement (C2088R) in *Lolium rigidum* could endow plants with resistance to the herbicides across the aryloxyphenoxypropionate (APP), cyclohexanedione (CHD), and phenylpyrazoline (PPZ) chemical groups [16]. The point mutation C2088R in *Lolium rigidum* corresponds to C2186R in rice (*Oryza sativa*), which is our target site *OsACC*-T1. Examination of 160 pH-PABE-7-esgRNA-transformed lines revealed that 33 harbored at least one T to C substitution in the target region (mutation efficiency of 20.6%) (Table 1; Additional file 2: Table S1). One of the mutant lines contained a homozygous substitution (T_4_T_7_ > C_4_C_7_), whereas the remaining 32 contained heterozygous substitutions: 20 with double-base substitutions (T_4_T_7_ > C_4_C_7_), ten with T_4_ > C_4_ single-base substitutions, and two that contained single-base substitutions providing the desired C2186R amino acid substitution at one of the alleles (T_7_ > C_7_; T0–7 and T0–13) (Fig. 2e; Table 1; Additional file 2: Figure S5b). We did not detect mutations in the potential off-target regions among all *OsACC*-T1 mutant lines (Additional file 2: Tables S2 and S3). We then assessed the herbicide resistance of the T0–13 mutant carrying the heterozygous C2186R substitution. After one week of growth on the regeneration medium supplemented with 0.086 ppm haloxyfop-R-methyl, the mutant plant had normal phenotypes with no symptoms of damage whereas wild-type (WT) plants displayed severe stunting and withered leaves (Fig. 2e). To the best of our knowledge, this is the first report of producing C2186R substitution of resistant rice plants using genome editing tools.

We also used the plant ABE system to generate base-edited plants in wheat by targeting *TaDEP1* and *TaGW2* genes. PABE-7 and pTaU6-esgRNA constructs (Additional file 2: Figure S1e and Table S1) were delivered into immature wheat embryos by particle bombardment, and plants regenerated without herbicide selection, as previously described [17]. Through T7E1 and Sanger sequencing, we obtained five A_8_ to G_8_ heterozygous *TaDEP1* mutant plants regenerated from 460 bombarded immature embryos (Table 1; Additional file 2: Figure S6a), with four mutants heterozygous for *TaDEP1-A* (*tadep1-AaBBDD*) and one mutant heterozygous for *TaDEP1-B* (*tadep1-AABbDD*) (Table 1; Additional file 2: Figure S6a). For the *TaGW2* target site, two heterozygous mutants were identified. Both harbored an A to G substitution at position 5 for *TaGW2-B* (*tagw2-AABbDD*) (Table 1; Additional file 2: Figure S6b). Again, no indels were observed in the target region of all mutant plants. Furthermore, PCR screening with six primer sets, specific for PABE-7 and pTaU6-esgRNA (Additional file 2: Figure S7a and Table S3), confirmed that three of five *TaDEP1* mutants and two *TaGW2* mutants did not carry the transgene vectors (Additional file 2: Figure S7b). Taken together, these results support that the plant ABE system is effective in inducing specific point mutations in rice and wheat in a highly specific and precise manner without causing other genomic modifications.

## Discussion

Despite the newly developed high efficiency of cytidine deaminase mediated C to T substitution exhibiting a great potential for disease therapeutic and agronomic traits engineering [4], additional base editing tools are needed for expanding editing more DNA nucleotides. Here, we adapted and optimized a plant ABE system (fusion of an evolved tRNA adenosine deaminase with nuclease-inactivated CRISPR/Cas9) to efficiently and specifically achieve targeted conversion of adenine to guanine in crop plants. To our knowledge, this is the first report of achieving wheat A to G base-edited plants and herbicide-resistant rice plants with the plant ABE system. High base-editing efficiency, low indels, and high purity products make this plant ABE system outperform HDR-mediated genome editing.

Based on the ABE7.10 architectures for human cells, we optimized the system for crop plants from two perspectives. One was by optimizing the position of the tRNA adenosine deaminase relative to the nCas9, and the number and locations of NLSs. Our observation shows that placing the ecTadA-ecTadA* adenosine deaminase at the N-terminus of nCas9 and the presence of three NLSs at the C-terminus (PABE-7) maximizes editing efficiency, probably because this configuration maximizes fusion protein folding and nuclear importing. The other improvement to the plant ABE system was based on comparing three forms of sgRNA (native sgRNA, esgRNA, and tRNA-sgRNA). We found that the esgRNA showed a higher editing efficiency than the native sgRNA and the tRNA-sgRNA in both protoplasts and regenerated plants, indicating that the esgRNA has a higher expression level and better binding activity with Cas9 [12]. With our most effective combination, PABE-7 plus esgRNA, we obtained base-edited rice and wheat plants in the T0 generation. The herbicide-resistant rice plants harboring the C2186R substitution in *OsACC* was also obtained*,* indicating this plant ABE system is a reliable tool for achieving targeted base editing in crop plants.

There are still opportunities for extending and optimizing the plant ABE system. One could use engineered Cas9 variants with different protospacer-adjacent motif (PAM) specificities (xCas9, SpCas9-VQR, SpCas9-VRER, SaCas9, and SaCas9-KKH), or Cpf1 [9, 18, 19], to expand the number of sites that can be targeted. The plant ABE system combined with the plant C to T base editing system by ligating sgRNA with different aptamers (MS2, PP7, COM, and boxB) [20, 21] could achieve simultaneous A to G and C to T changes, and could be used to correct point mutations related to important agronomic traits. It could also provide a novel forward genetics tool to screen gain-of-function and partial loss-of-function genetic variants at the resolution of single bases. Furthermore, plant ABE ribonucleoproteins (RNPs) could be delivered to create transgene-free mutant plants, which could avoid inserting recombinant DNA into host genomes, and would have a good chance of being commercialized [17, 22].

## Conclusions

We describe here an efficient plant base-editing system that induces precise A•T to G•C substitutions across a broad range of endogenous genomic loci. The effective deamination window of this plant ABE system extends from positions 4 to 8 of the protospacer and produces high-fidelity substitutions at the targeted loci with low indels. These findings, together with previously described plant substitution systems [23–26], extend the application of base editing to the majority of codons and now provides feasible opportunities for significant in vivo mutagenesis studies and trait improvement in plants.

## Methods

### Plasmid construction

To construct vectors PABE-1 to PABE-7, the tRNA editing deaminase ecTadA, ecTadA*, 32aa linker, and nCas9 (D10A) sequences were codon-optimized for cereal plants, and synthesized commercially (GENEWIZ, Suzhou, China). The various combinations of the ecTadA-ecTadA* and nCas9 fusion protein sequences were cloned into the vector pJIT163 backbone. The native constructs pOsU3-sgRNA and pTaU6-sgRNA, tRNA-sgRNA of pOsU3-tRNA-sgRNA and pTaU6-tRNA-sgRNA were made as previously described [14, 27, 28]; the esgRNA of pOsU3-esgRNA and pTaU6-esgRNA were synthesized commercially (GENEWIZ, Suzhou, China). To construct the pH-PABE-7-esgRNA and pH-PABE-7-sgRNA binary vector, PABE-7 and esgRNA or sgRNA expression cassettes were cloned into the pHUE411 backbone [29]. Point mutations were introduced into the coding sequence of GFP with the Fast Mutagenesis System (TransGen Biotech, Beijing, China), yielding expression cassettes producing mGFP. All the primer sets used in this work are listed in Additional file 2: Table S3 and were synthesized by Beijing Genomics Institute (BGI).

### Protoplast transfection

We used the winter wheat variety Kenong199 and the *Japonica* rice variety Zhonghua11 to prepare the protoplasts used in this study. Protoplast isolation and transformation were performed as previously described [27, 28]. Plasmid DNA (10 μg per construct) was introduced into the desired protoplasts by PEG-mediated transfection, the mean transformation efficiency being 45–60% by flow cytometry (FCM). The transfected protoplasts were incubated at 23 °C. At 60 h post-transfection, the protoplasts were collected to extract genomic DNA for deep amplicon sequencing and T7E1 and PCR restriction enzyme digestion assays (PCR-RE assays; see below).

### DNA extraction

Genomic DNA was extracted with a DNA quick Plant System (TIANGEN BIOTECH, Beijing, China). The targeted site was amplified with specific primers, and the amplicons were purified with an EasyPure PCR Purification Kit (TransGen Biotech, Beijing, China), and quantified with a NanoDrop™ 2000 Spectrophotometer (Thermo Fisher Scientific, Waltham, MA, USA).

### Next-generation sequencing

Genomic DNA extracted from the desired protoplast samples at 60 h post-transfection was used as template. In the first round PCR, the target region was amplified using site-specific primers (Additional file 2: Table S3). In the second round PCR, both forward and reverse barcodes were added to the ends of the PCR products for library construction (Additional file 2: Table S3). Equal amounts of the PCR products were pooled and samples were sequenced commercially (Mega Genomics, Beijing, China) using the Illumina NextSeq 500 platform. The sgRNA target sites in the sequenced reads were examined for A to G substitutions and indels. The amplicon sequencing was repeated three times for each target site, using genomic DNA extracted from three independent protoplast samples.

### *Agrobacterium*-mediated transformation of rice callus cells

*Agrobacterium tumefaciens* strain AGL1 was transformed with the pH-PABE-7-esgRNA or pH-PABE-7-sgRNA binary vectors by electroporation. *Agrobacterium*-mediated transformation of callus cells of Zhonghua11 was conducted as reported [30]. Hygromycin (50 μg/ml) was used to select transgenic plants.

### Biolistic delivery of DNA constructs into wheat immature embryo cells

The plasmid DNAs of PABE-7 and pTaU6-esgRNA were simultaneously delivered into the immature embryos of Kenong199 via particle bombardment as previously described [17]. After the bombardment, the embryos were cultured for plantlet regeneration on the media without a selective agent [17].

### Mutant identification by T7E1 and PCR-RE assays and Sanger sequencing

T7E1 and PCR-RE assays and Sanger sequencing were performed to identify rice and wheat mutants with A to G conversions in target regions, as described previously [27, 28]. For rice, the T0 transgenic plants were examined individually. For wheat, plantlets (usually 3–4) derived from each bombarded immature embryo were pooled for the assays, and the positive pools were examined further to identify individual mutant plantlets [28]. A to G mutation frequencies were calculated from band intensities measured with UVP VisionWorks LS Image Acquisition Analysis Software 7.0, as described [27].

### Detection of off-target mutations

Likely off-targets were predicting using the online tool CRISPR-P [31]. The off-target sites in *OsACC*-T1 in the rice genome were identified and examined in this study.

## Additional files

Additional file 1: **Sequences** Complete coding sequences of the PABE-1 to PABE-7 fusion cistrons optimized in this study. (DOCX 4108 kb)

Additional file 2: **Figure S1.** The sequences of the sgRNA expression vectors for rice and wheat. **Figure S2.** Product purity of plant ABE for rice genomic sites. **Figure S3.** Product purity of plant ABE for wheat genomic sites. **Figure S4.** The effect of spacer length of esgRNA on indel efficiency. **Figure S5.** Identification and analysis of the rice plantlets with targeted A to G conversions by pH-PABE-7-esgRNA. **Figure S6.** Identification and analysis of the wheat plantlets with targeted A to G conversions by PABE-7. **Figure S7.** Constructs used for base editing of *TaDEP1* and *TaGW2* and detection of transgene integration in the resultant T0 mutants. **Table S1.** Description of sgRNA target sites and sequences. **Table S2.** Potential off-target sites analyzed for OsACC-T1 endogenous genomic loci. **Table S3.** PCR primers used in this study. (DOC 6095 kb)
