# Supplementary material for: Expanded base editing in rice and wheat using a Cas9-adenosine deaminase fusion
Source: Genome Biol. 2018 May 29;19:59. doi: 10.1186/s13059-018-1443-z (PMC5972399; doi:10.1186/s13059-018-1443-z)

**Sequences.** Complete coding sequences of the PABE-1 to PABE-7 fusion cistrons optimized in this study. The NLSs are written in lower cases. The codon-optimized ecTadA, 32aa linker and ecTadA* are highlighted in green, blue and brown, respectively. The codon-optimized nCas9 (D10A) is shown in bold.


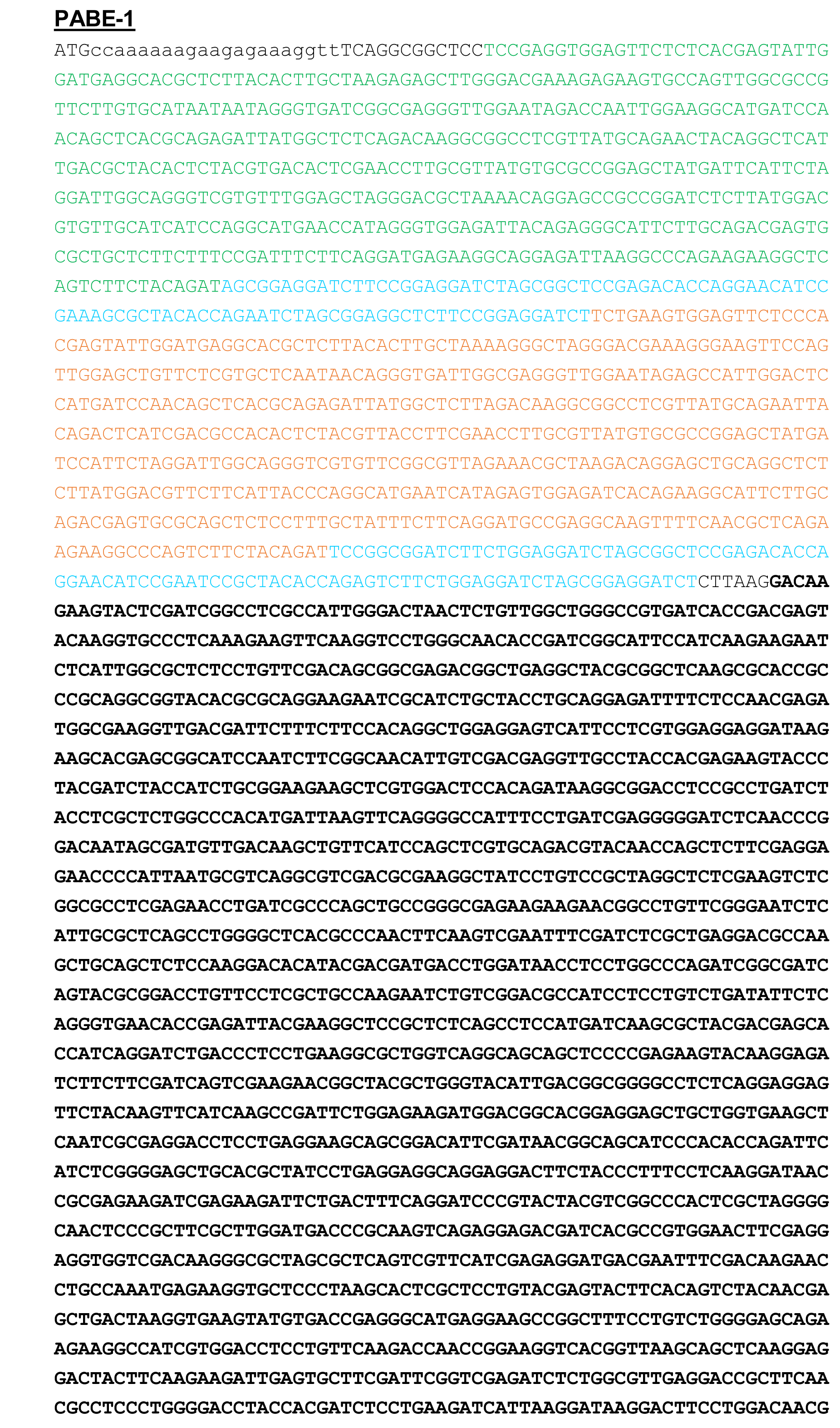


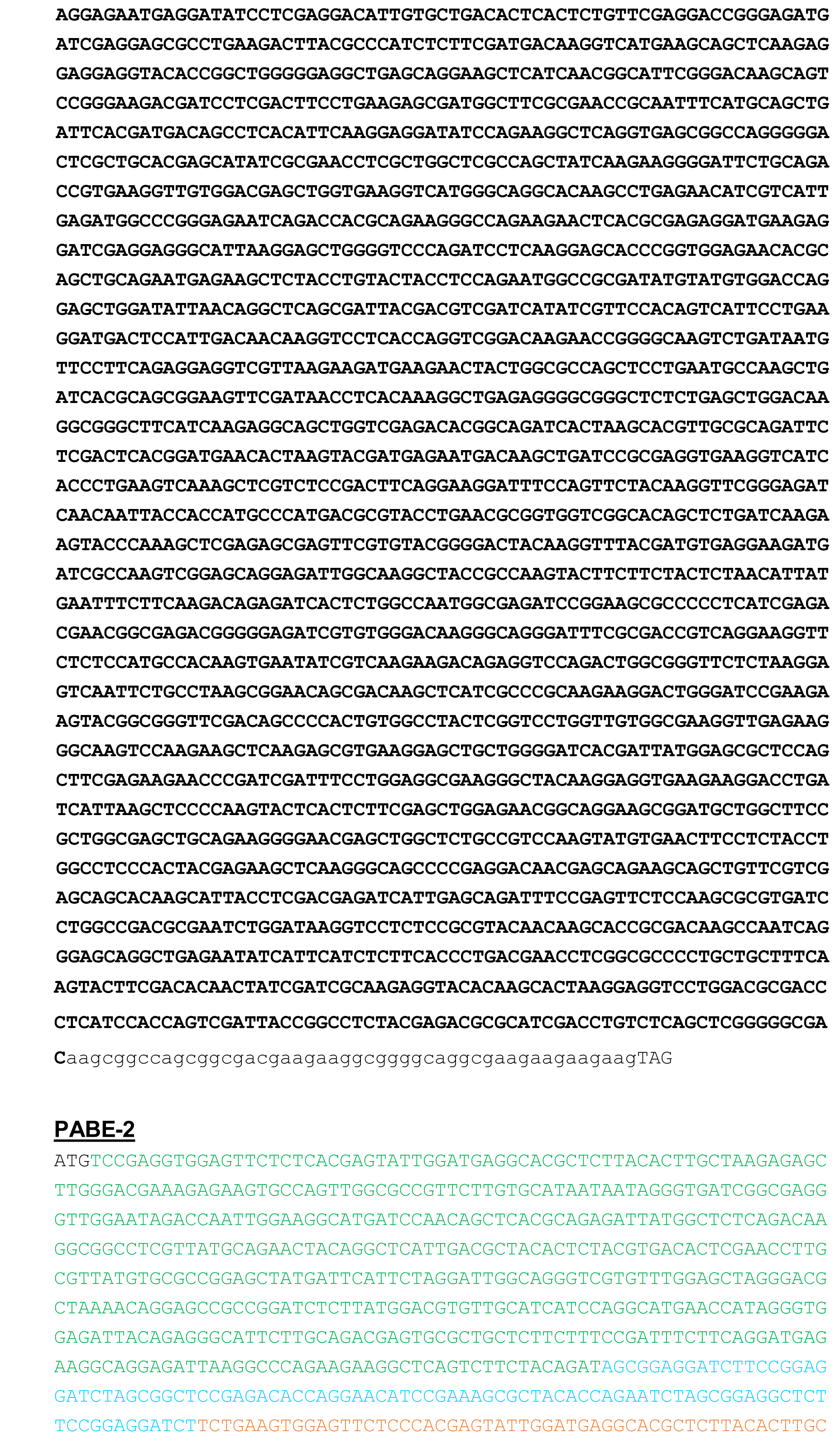


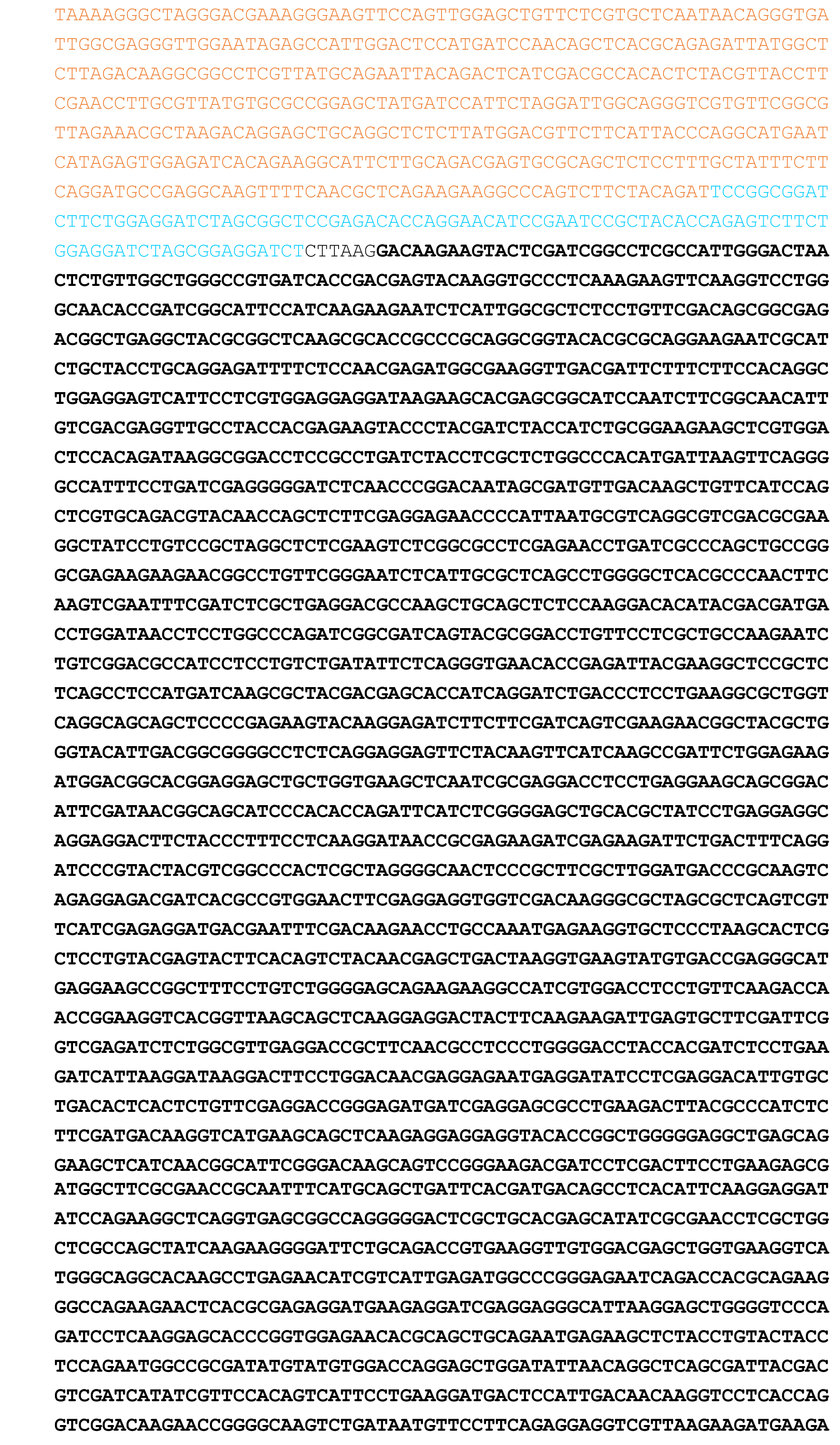


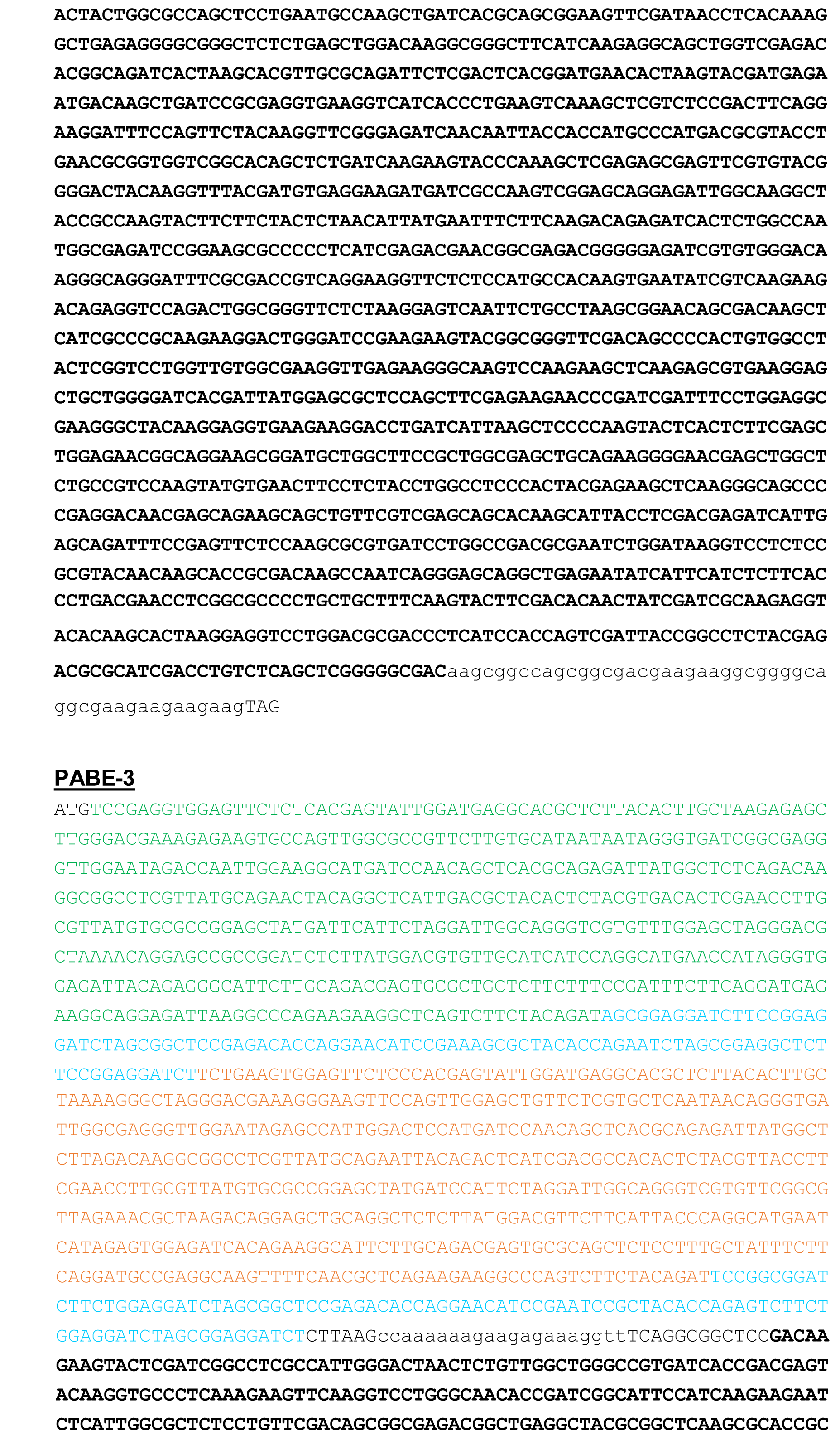


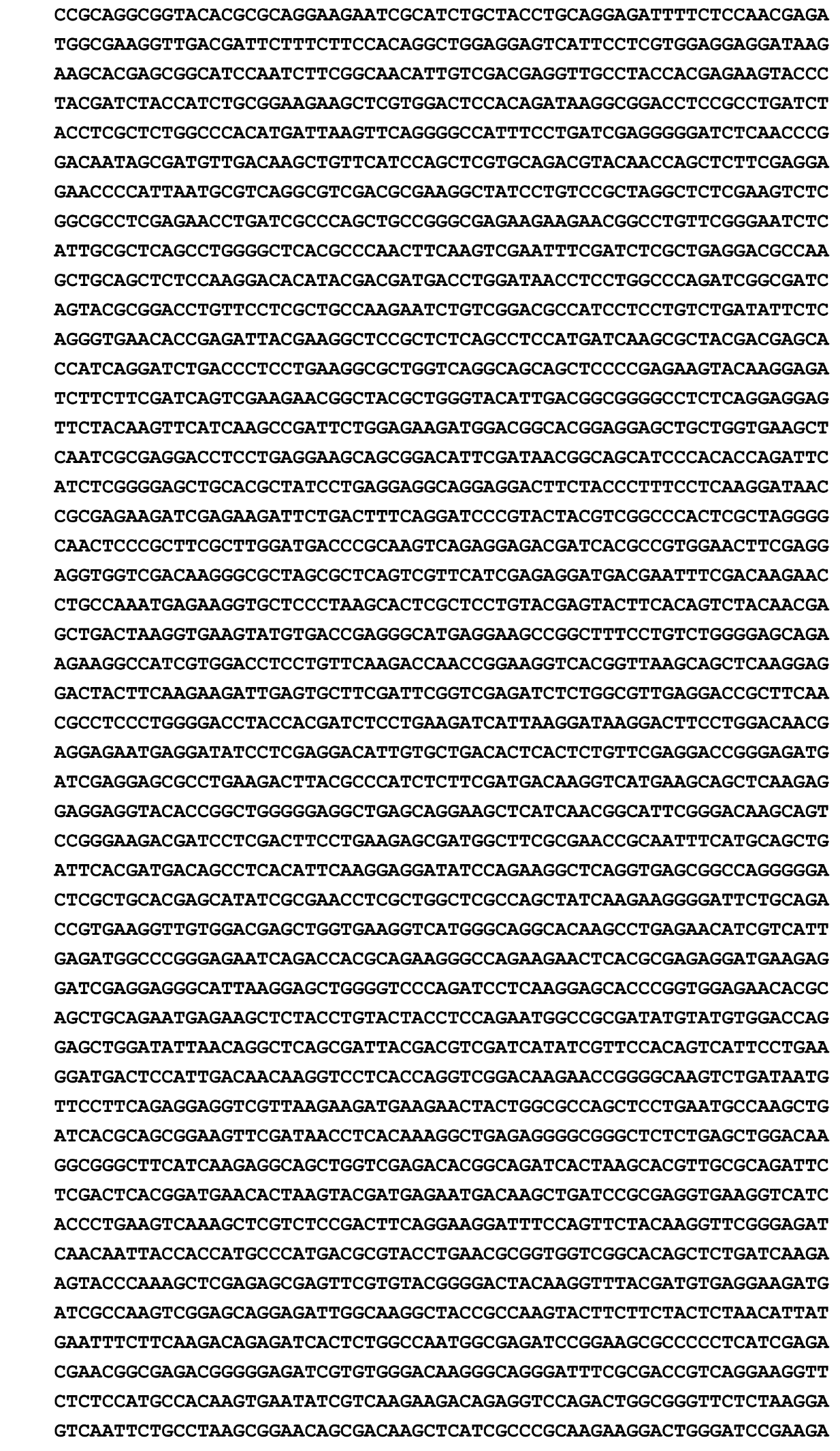


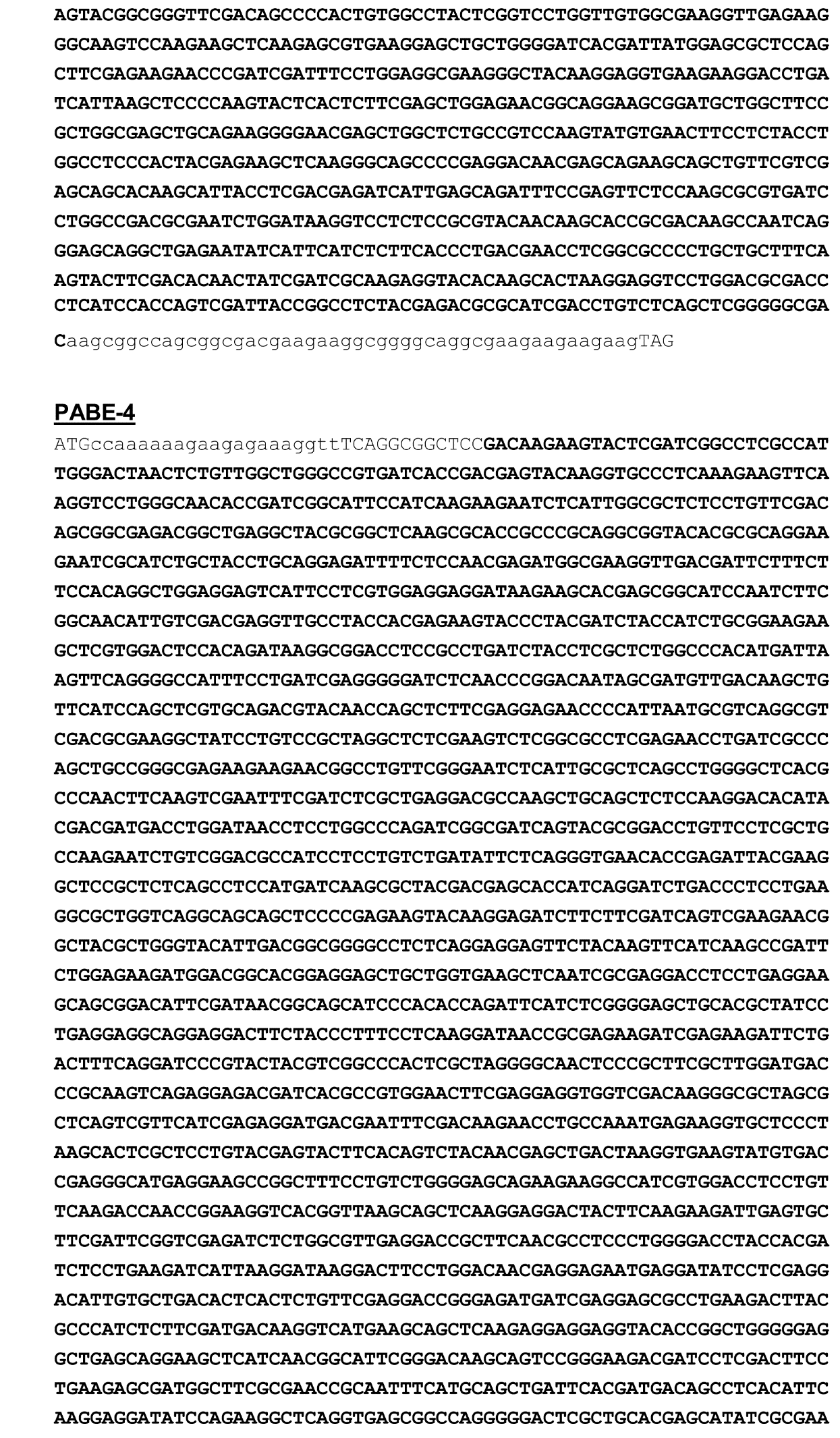


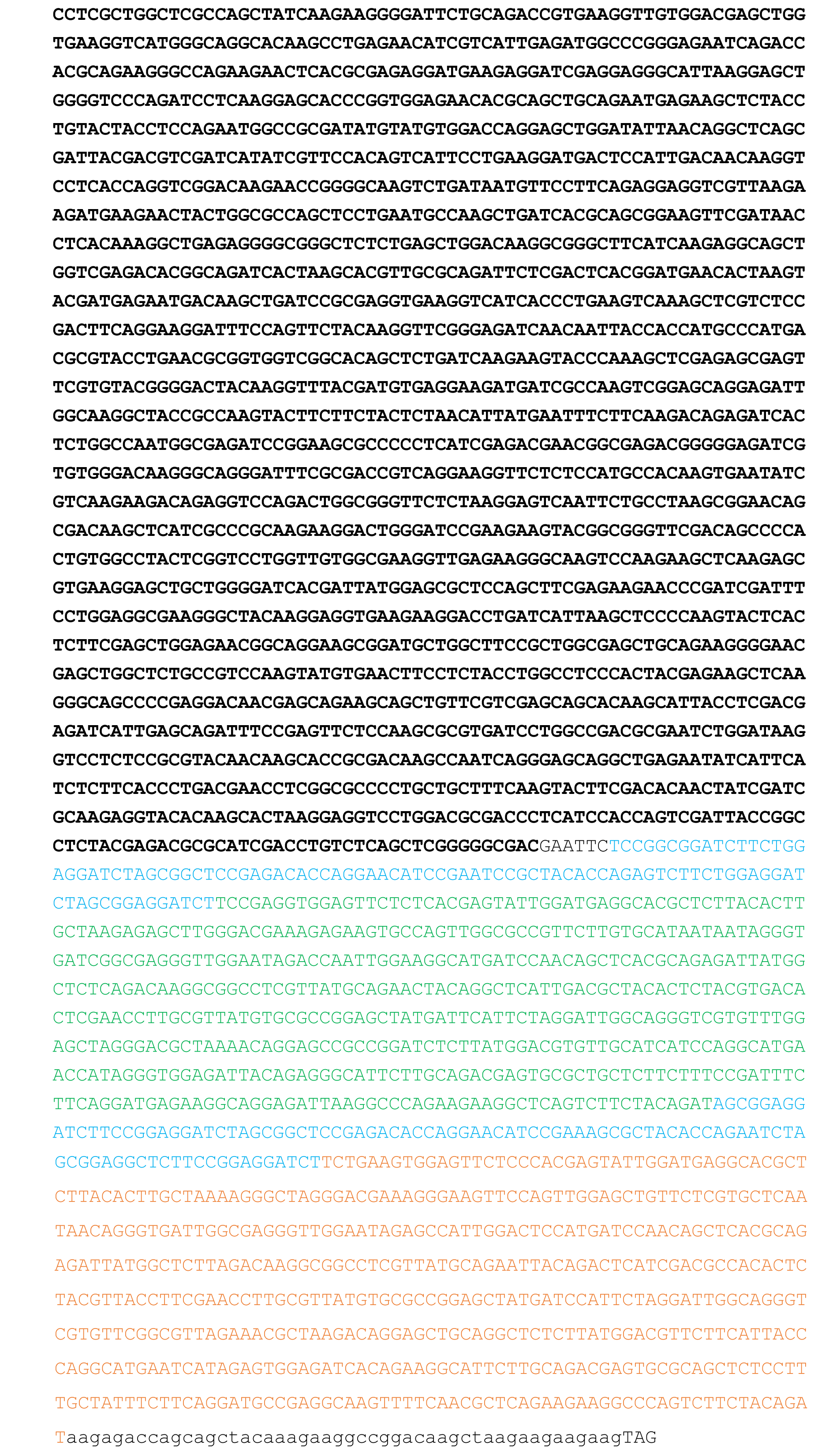


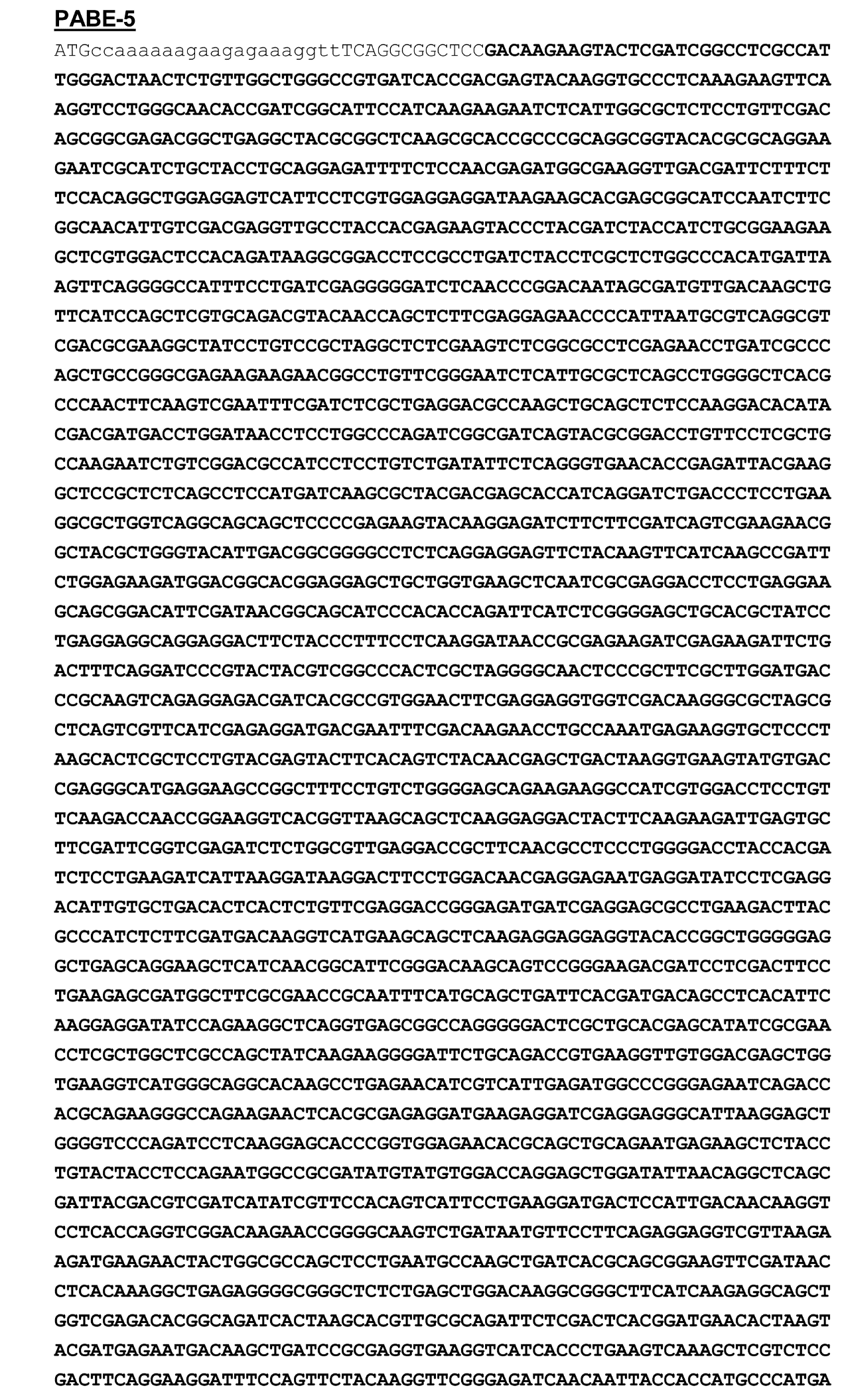


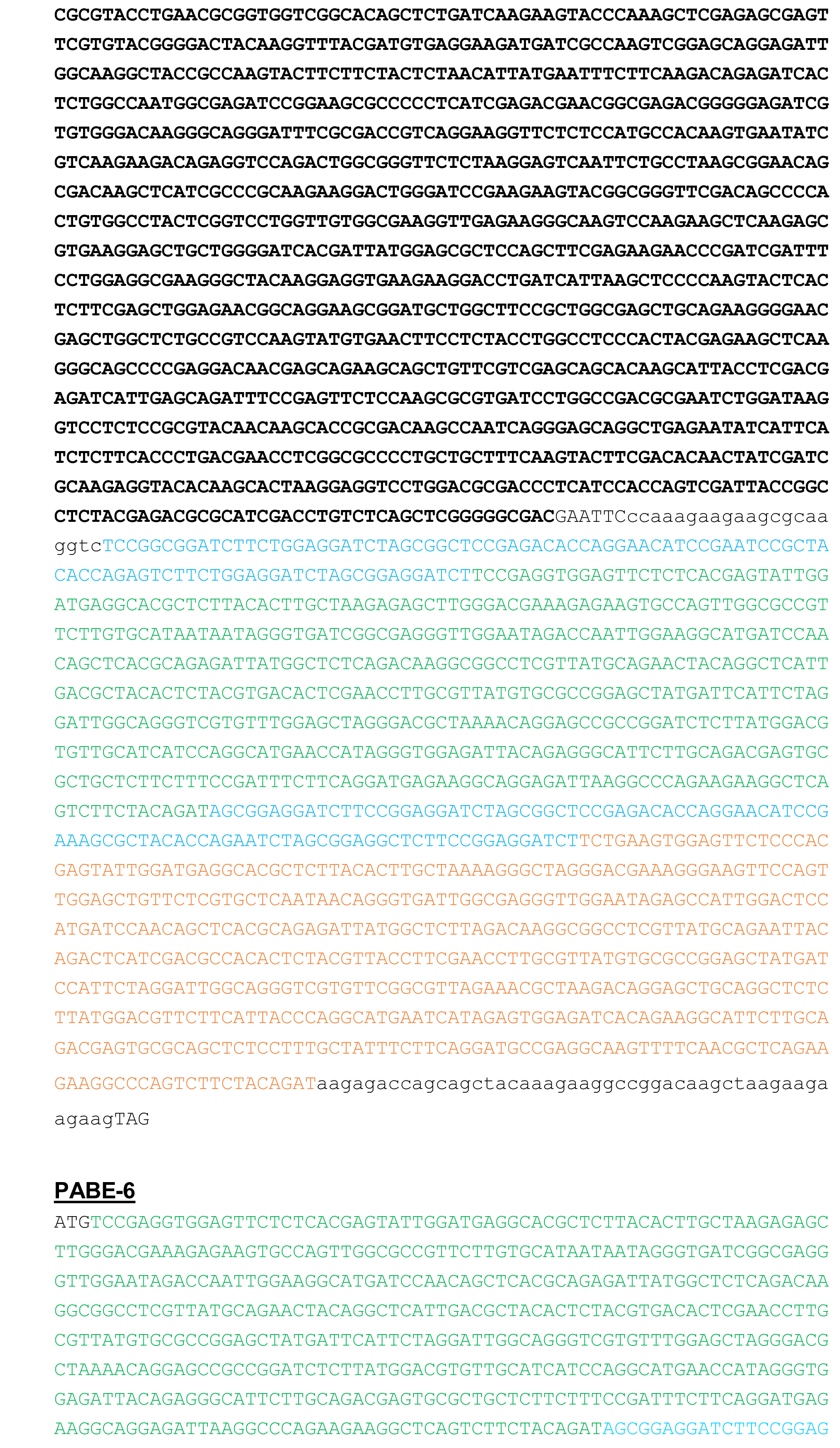


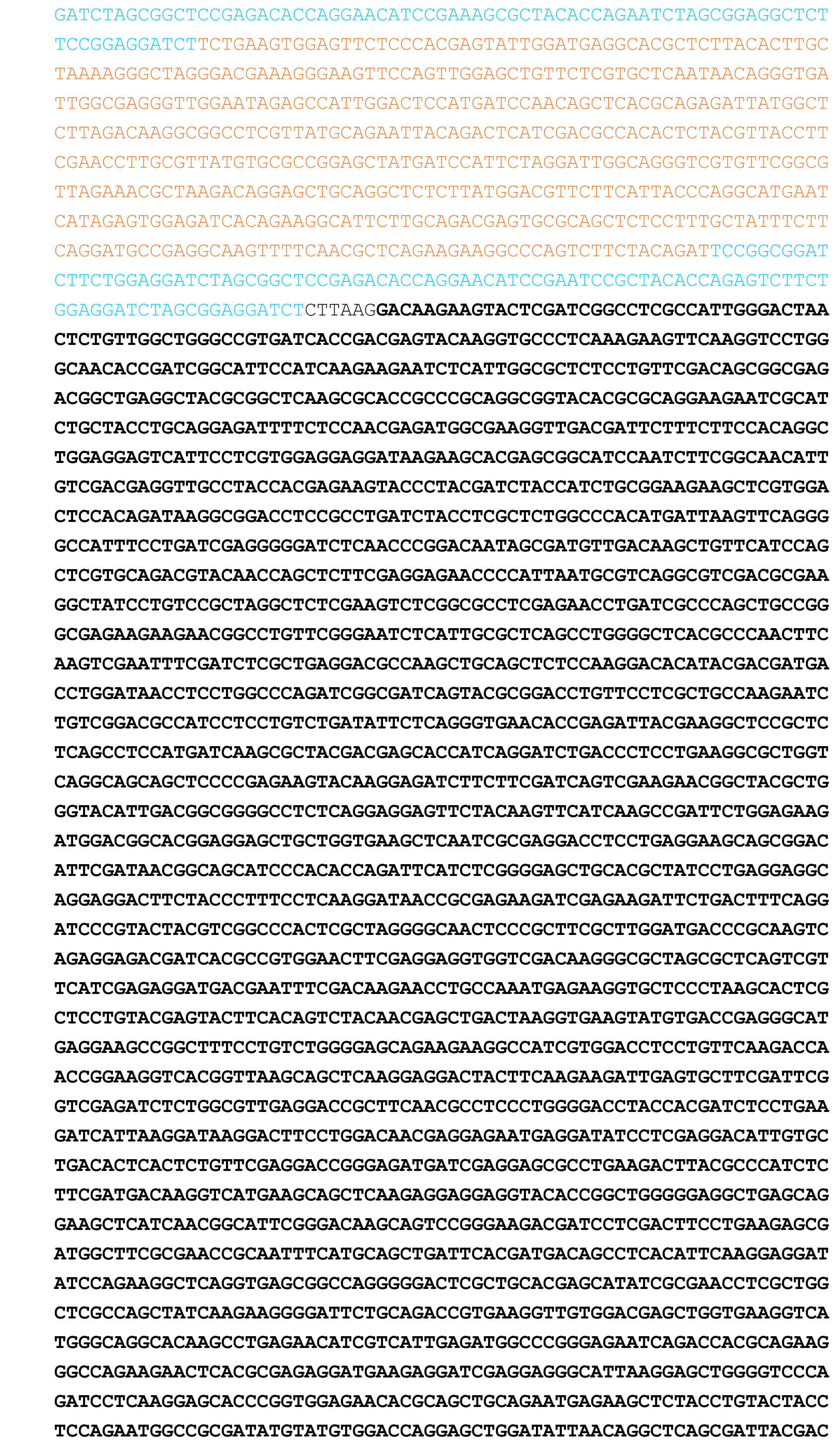


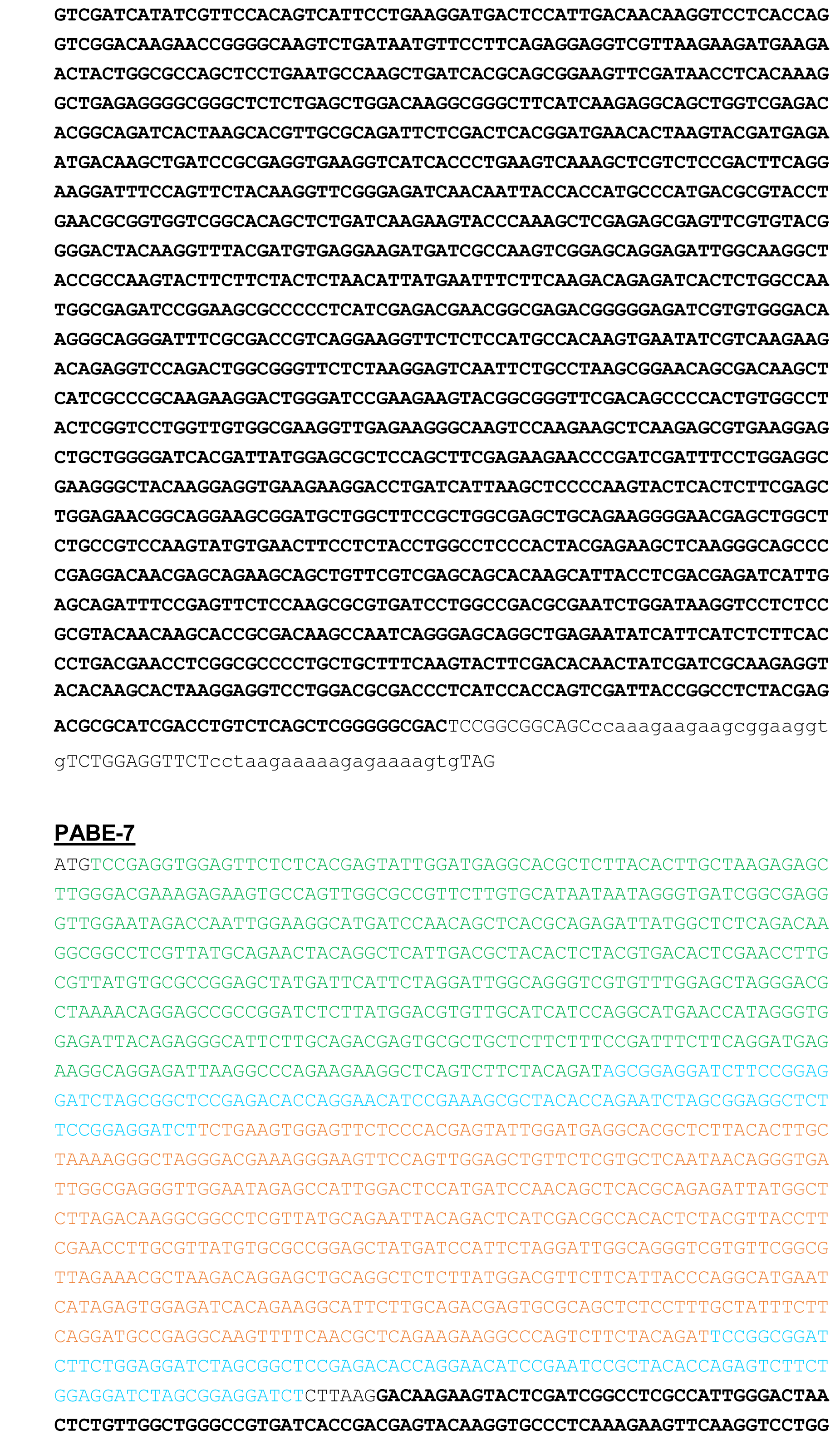


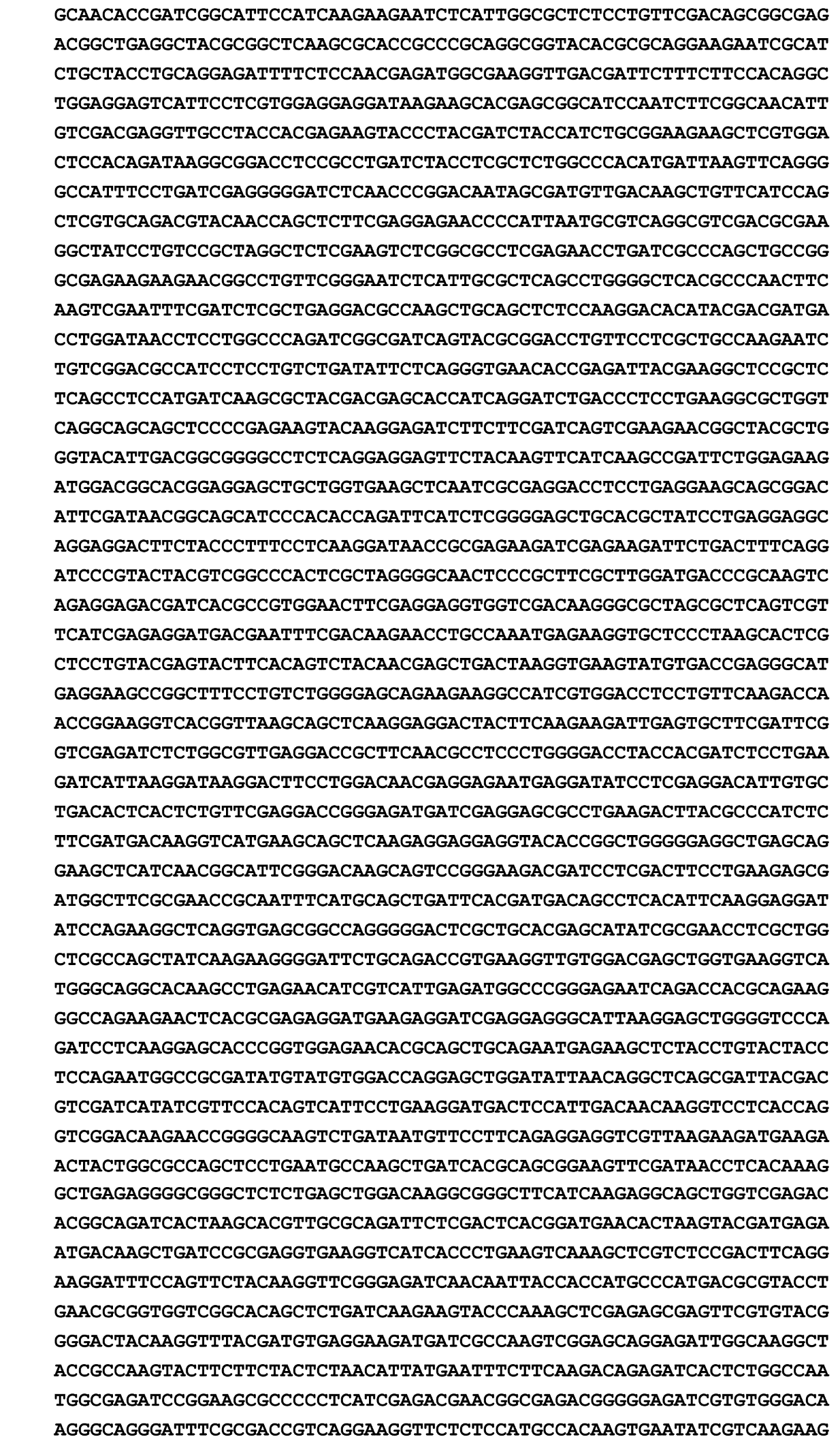


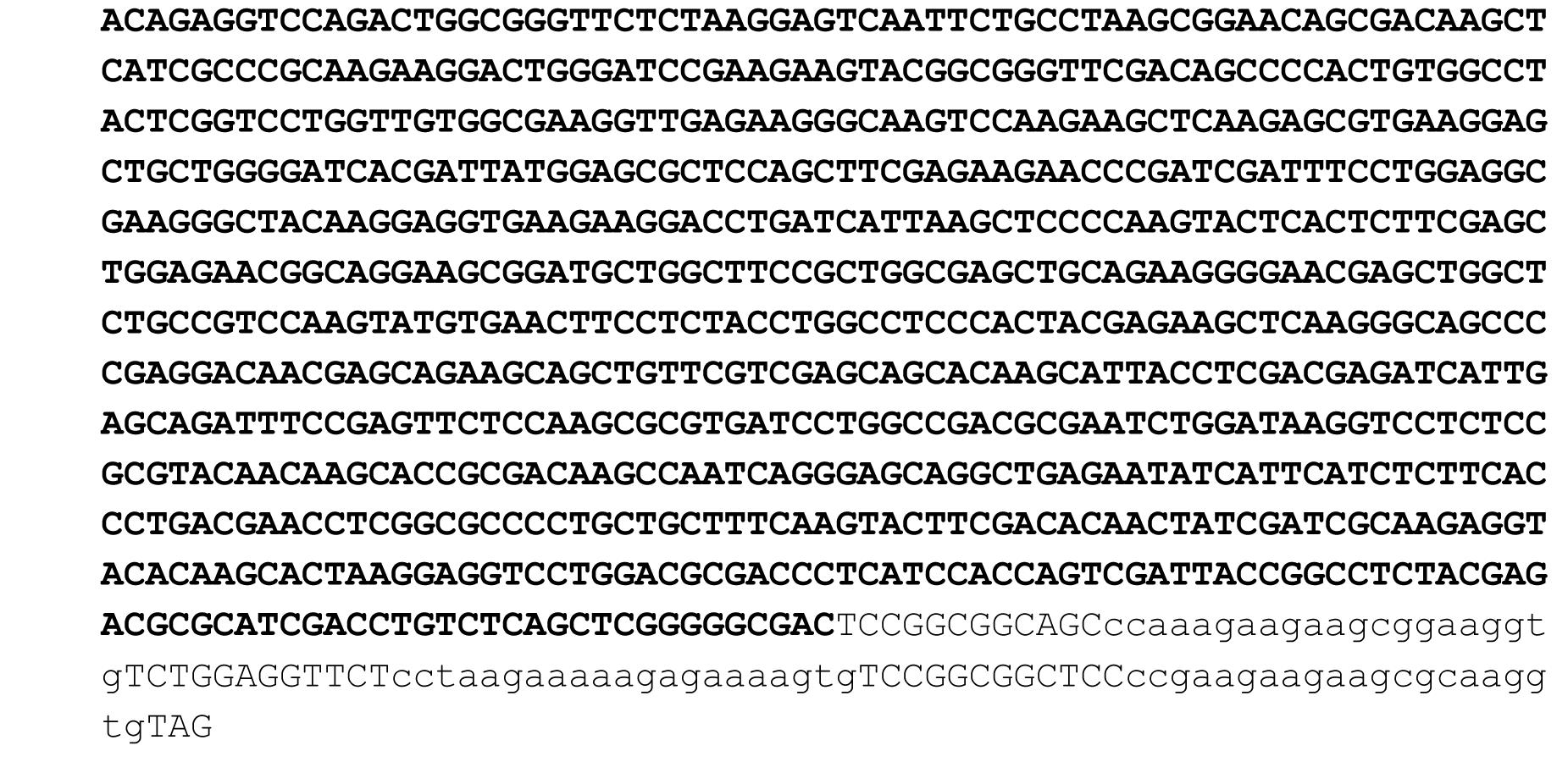

Supplement: Supplementary file 1 — Sequences Complete coding sequences of the PABE-1 to PABE-7 fusion cistrons optimized in this study. (DOCX 4108 kb) [file 13059_2018_1443_MOESM1_ESM.docx]
