# Supplementary material for: Expanded base editing in rice and wheat using a Cas9-adenosine deaminase fusion
Source: Genome Biol. 2018 May 29;19:59. doi: 10.1186/s13059-018-1443-z (PMC5972399; doi:10.1186/s13059-018-1443-z)
Supplement: Supplementary file 2 — Figure S1. The sequences of the sgRNA expression vectors for rice and wheat. Figure S2. Product purity of plant ABE for rice genomic sites. Figure S3. Product purity of plant ABE for wheat genomic sites. Figure S4. The effect of spacer length of esgRNA on indel efficiency. Figure S5. Identification and analysis of the rice plantlets with targeted A to G conversions by pH-PABE-7-esgRNA. Figure S6. Identification and analysis of the wheat plantlets with targeted A to G conversions by PABE-7. Figure S7. Constructs used for base editing of TaDEP1 and TaGW2 and detection of transgene integration in the resultant T0 mutants. Table S1. Description of sgRNA target sites and sequences. Table S2. Potential off-target sites analyzed for OsACC-T1 endogenous genomic loci. Table S3. PCR primers used in this study. (DOC 6095 kb) [file 13059_2018_1443_MOESM2_ESM.doc]

**
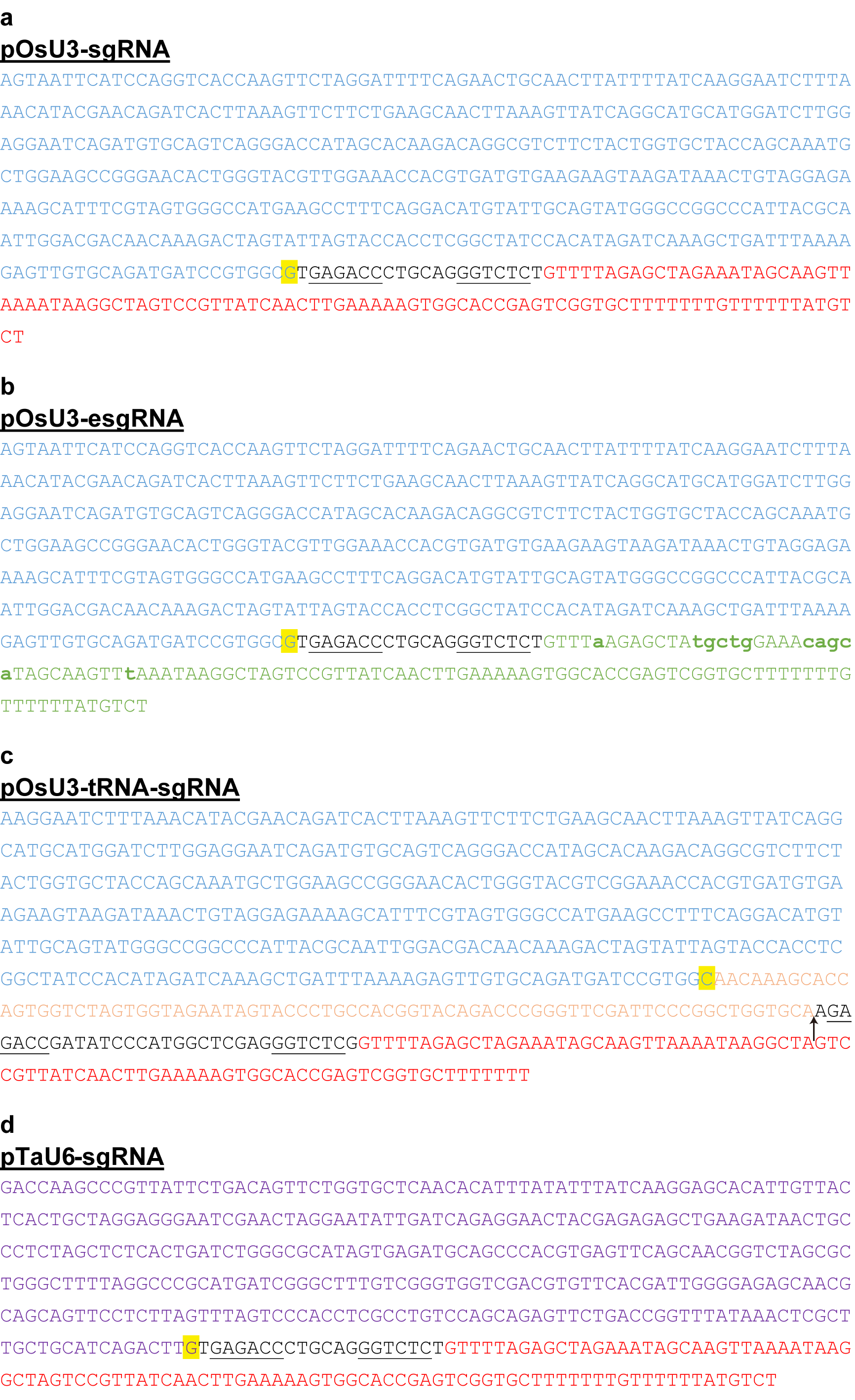
**

**
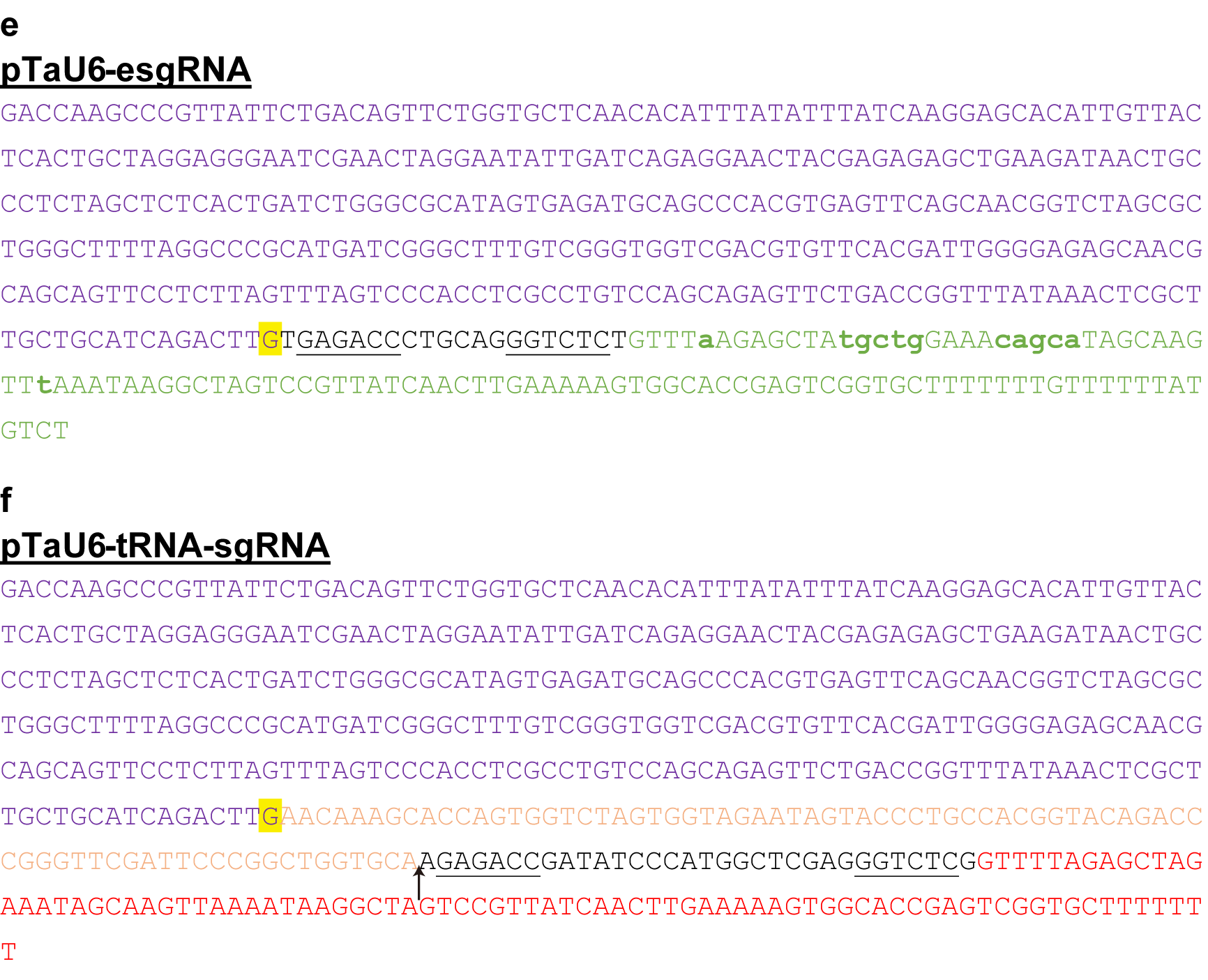
**

**Figure S1.** The sequences of the sgRNA expression vectors for rice and wheat. pOsU3-sgRNA (**a**), pOsU3-esgRNA (**b**), pOsU3-tRNA-sgRNA (**c**), pTaU6-sgRNA (**d**), pTaU6-esgRNA (**e**), pTaU6-tRNA-sgRNA (**f**). The rice U3 promoter, wheat U6 promoter, sgRNA scaffold, esgRNA scaffold and tRNA sequences are highlighted in blue, purple, red, green and brown, respectively. The transcription initiation sites of rice U3 promoter and wheat U6 promoter are indicated in yellow background. The black arrow indicates RNase Z cleavage site of tRNA. Two BsaI sites are underlined. The guide sequence can be inserted between the two BsaI sites using annealed oligos.

**
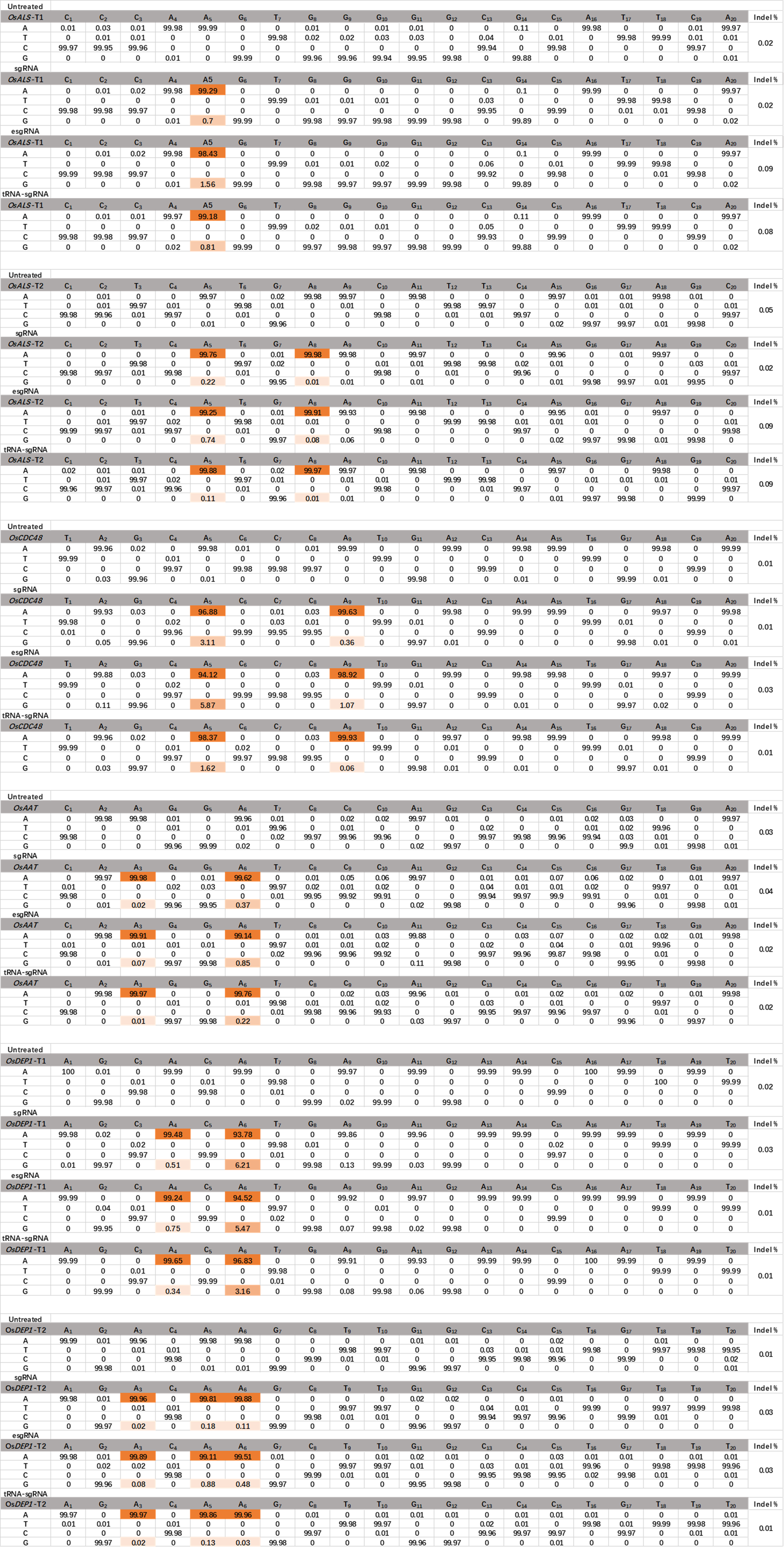
**

**
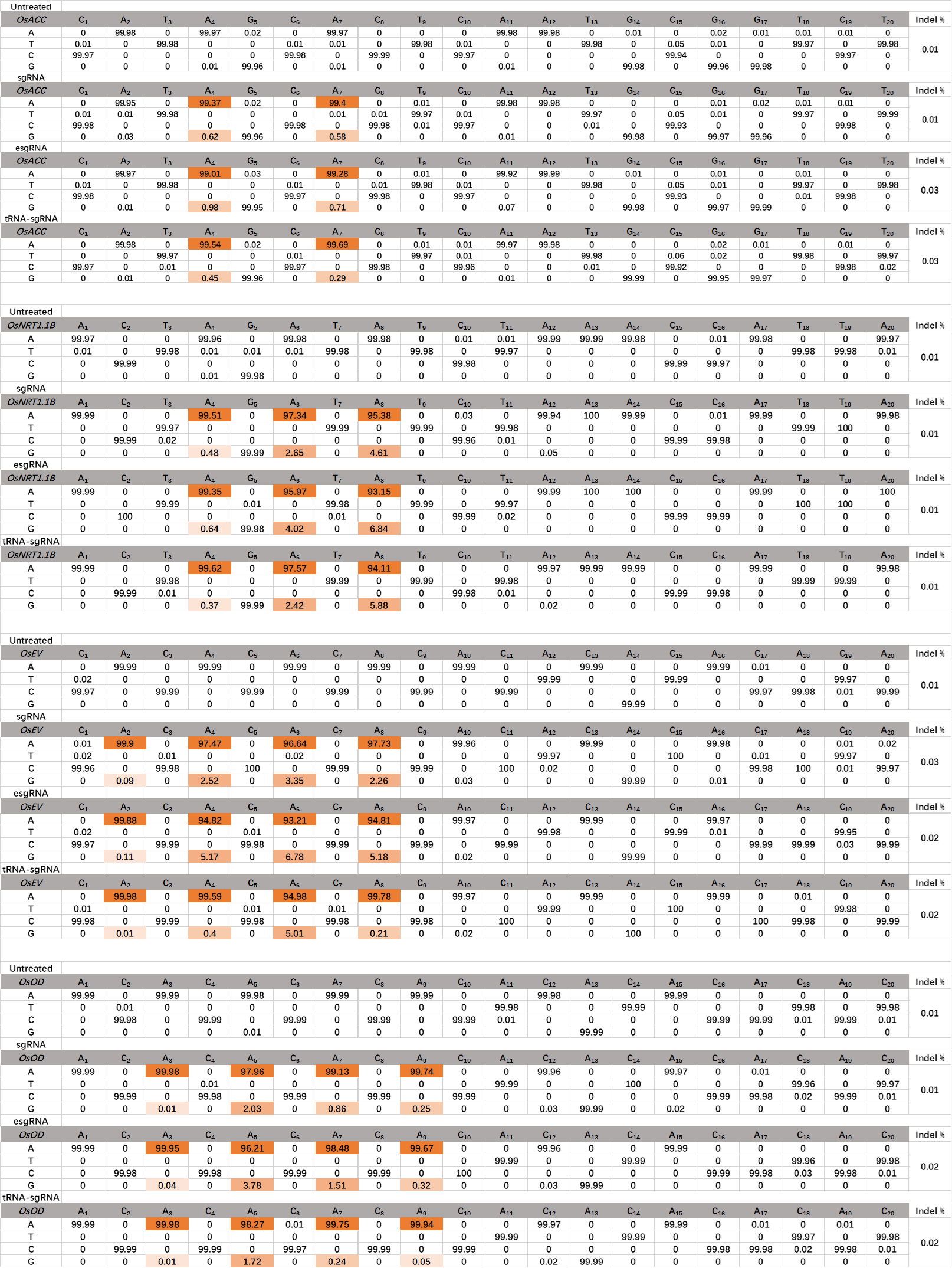
**

**Figure S2.** Product purity of plant ABE for rice genomic sites. Product distributions and indel frequencies at ten representative rice genomic DNA sites in rice protoplasts treated with PABE-7 and the corresponding native sgRNA, esgRNA and tRNA-sgRNA. A total of 48,616-111,697 sequencing reads were used at every position.


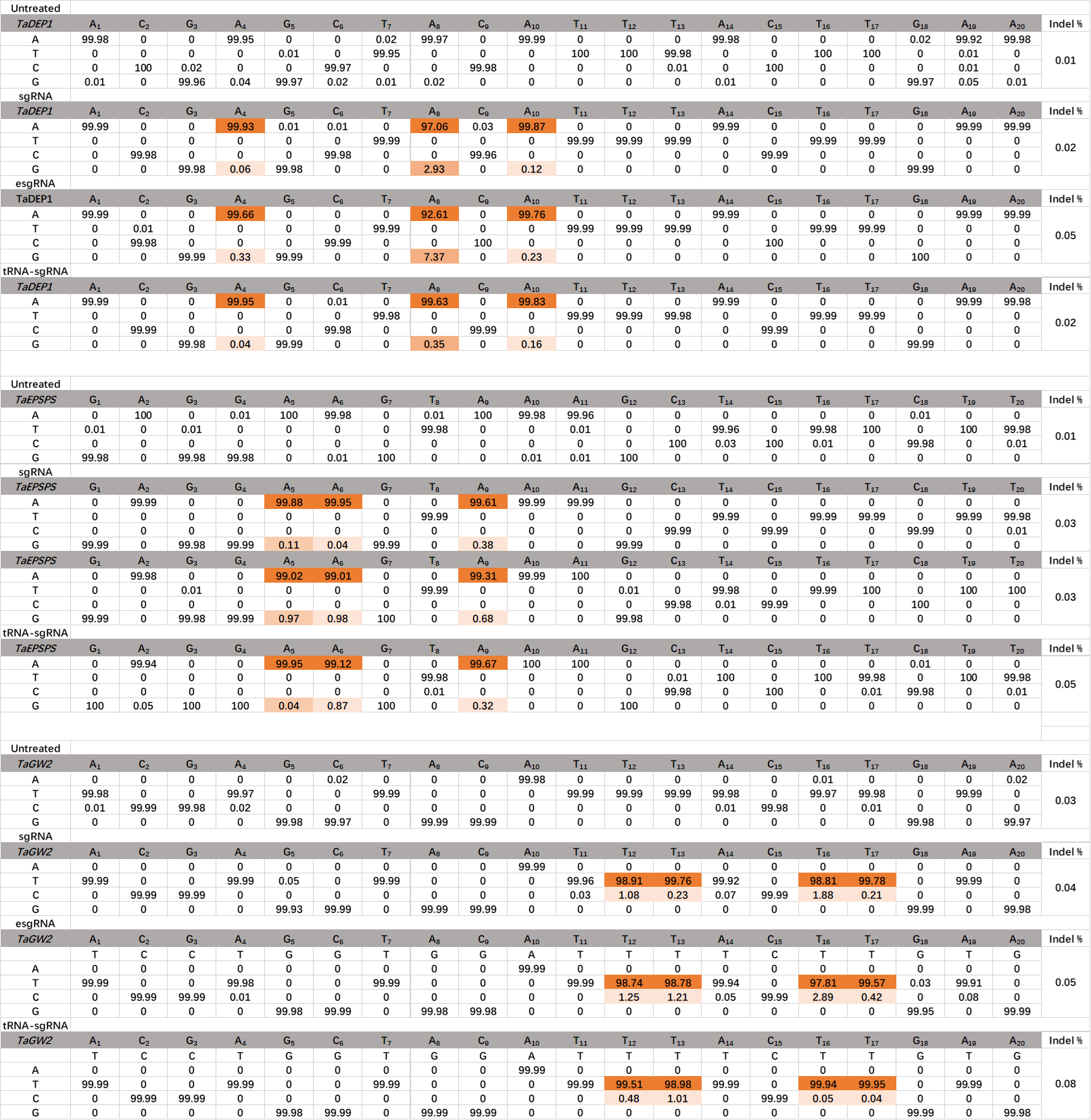


**Figure S3.** Product purity of plant ABE for wheat genomic sites. Product distributions and indel frequencies at three representative wheat genomic DNA sites in wheat protoplasts treated with PABE-7 and the corresponding native sgRNA, esgRNA and tRNA-sgRNA. A total of 28,110-28,4527 sequencing reads were used at every position.


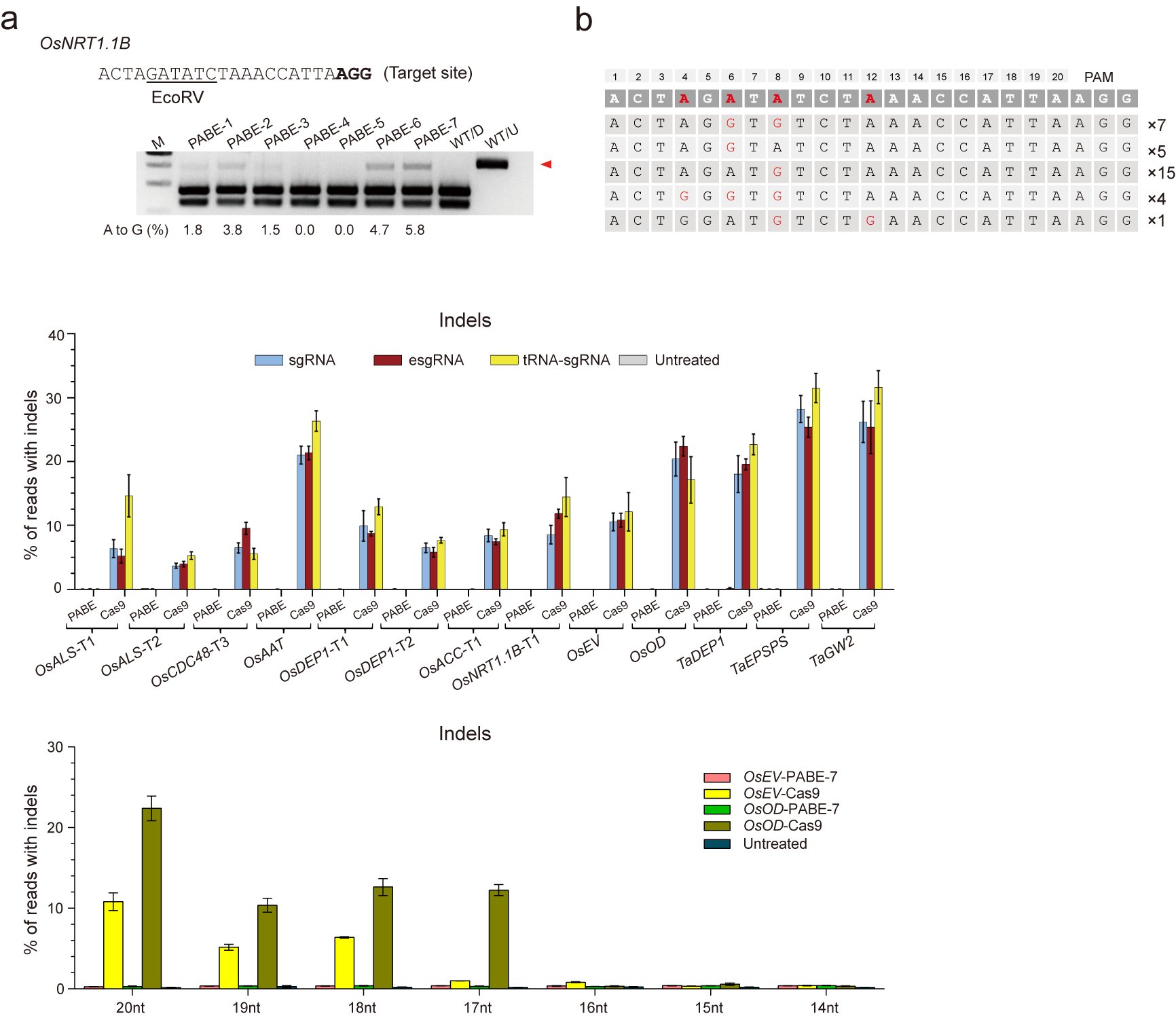


**Figure S4.** The effect of spacer length of esgRNA on indel efficiency. Indel frequencies induced by the PABE-7 or Cas9 with esgRNAs of different length varying from 14 to 20-nt were determined at the indicated editing positions. An untreated protoplast sample was used as control. Each frequency (mean±s.e.m.) was calculated using the data from three independent biological replicates (*n*=3).


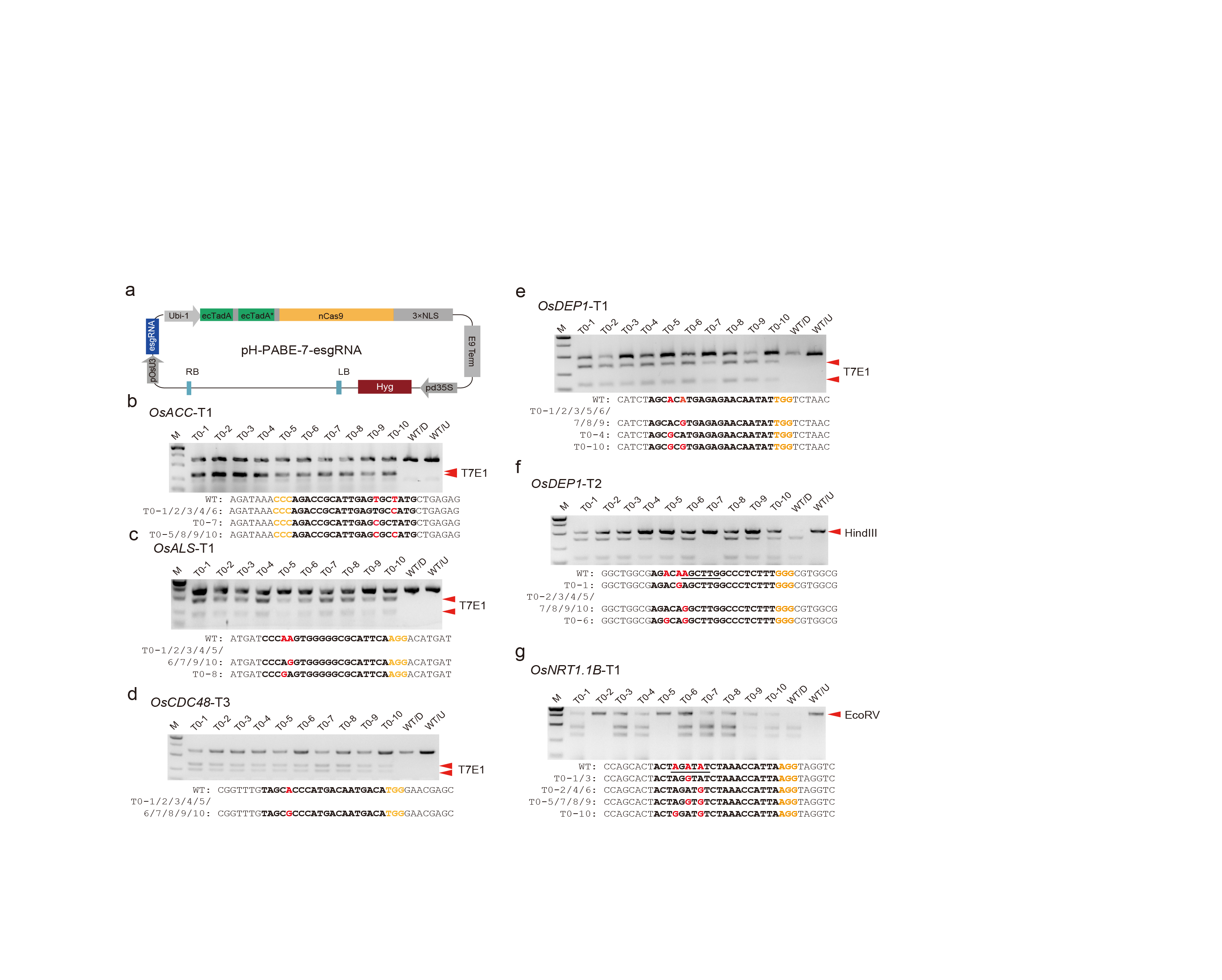


**Figure S5.** Identification and analysis of the rice plantlets with targeted A to G conversions by pH-PABE-7-esgRNA. **(a)** Schematic of the T-DNA expression vector pH-PABE-7-esgRNA for targeting *OsACC*-T1, *OsALS*-T1, *OsCDC48*-T3, *OsDEP1*-T1, *OsDEP1*-T2, and *OsNRT1.1B-*T1 genes. The coding sequence of hygromycin B phosphotransferase (Hyg, shown in red) is driven by pd35S promoter. (**b, c, d, e, f, g**) Outcome of T7E1 and PCR-RE assays for *OsACC*-T1 (**b**), *OsALS*-T1 (**c**)*, OsCDC48*-T3 (**d**), *OsDEP1*-T1 (**e**), *OsDEP1*-T2 (**f**) and *OsNRT1.1B-*T1 (**g**) mutants. The A/T bases targeted are highlighted in red. The PAM sequence is shown in brown. The HindIII or EcoRV restriction sites are underlined. Ten T0 plantlets (T0-1 to T0-10) were analyzed. WT/D and WT/U indicate the genomic DNA amplicons of wild type (WT) control with or without the digestion by T7E1 or restriction enzyme. Thirty-three mutants, forty-two mutants, sixty mutants, eighty-three mutants, thirty-four mutants and one hundred and forty-nine mutants were identified for *OsACC*-T1, *OsALS*-T1, *OsCDC48*-T3, *OsDEP1*-T1, *OsDEP1*-T2, and *OsNRT1.1B-*T1 respectively. The bands marked by red arrowheads are diagnostic of positive base editing. The sequences were determined by Sanger sequencing.


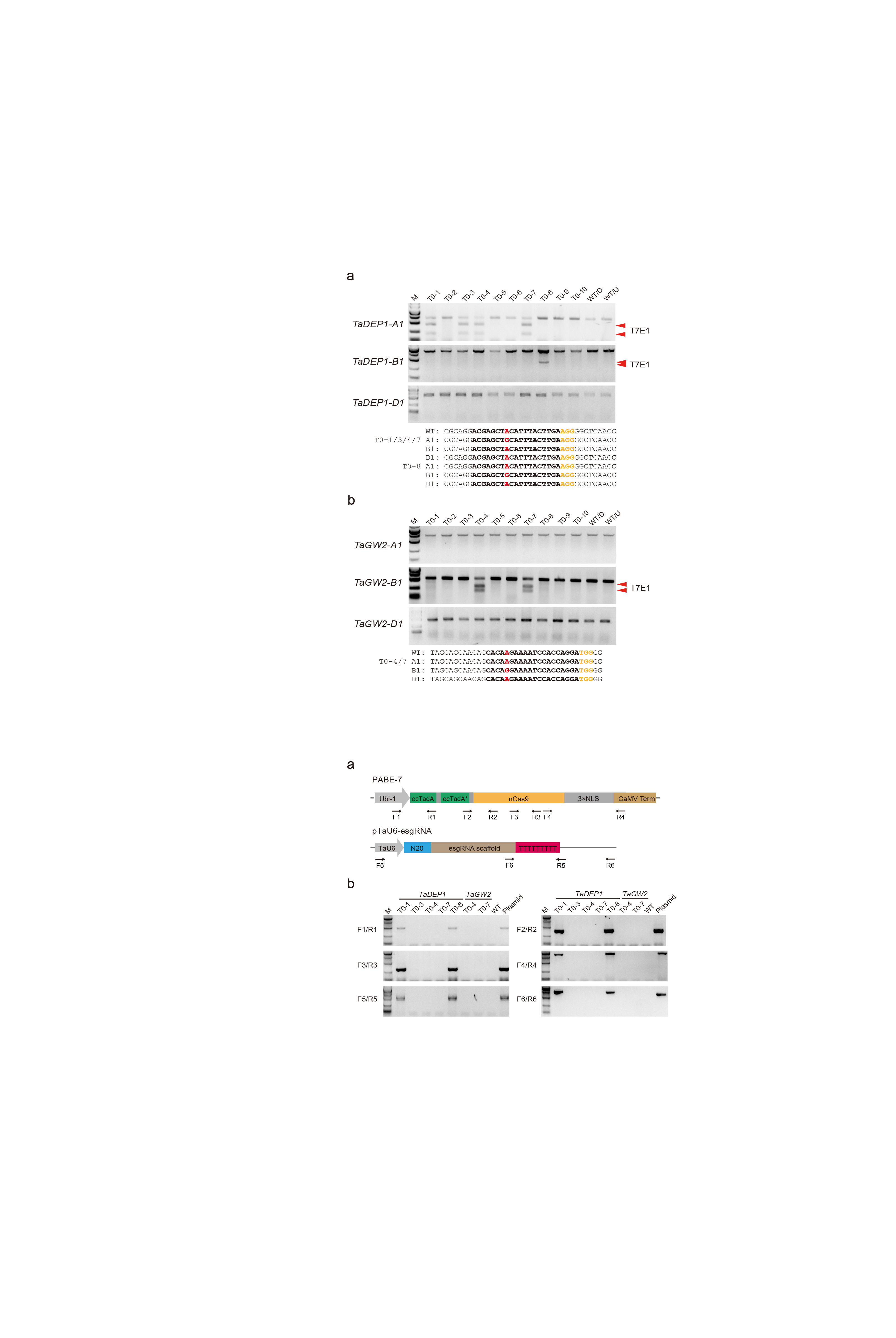


**Figure S6.** Identification and analysis of the wheat plantlets with targeted A to G conversions by PABE-7. **(a, b**) The results of T7E1 assays for *TaDEP1* (**a**) and *TaGW2* (**b**) mutants. The A bases targeted are highlighted in red. The PAM sequence is shown in brown. Ten T0 plantlets (T0-1 to T0-10) were analyzed. WT/D and WT/U indicate the genomic DNA amplicons of wild type (WT) control with or without the digestion by T7E1. Five heterozygous mutants (T0-1, T0-3, T0-4, T0-7 and T0-8) and two heterozygous mutants (T0-4 and T0-7) were identified for *TaDEP1* and *TaGW2* respectively. The bands marked by red arrowheads are diagnostic of positive base editing. The sequences were determined by Sanger sequencing.


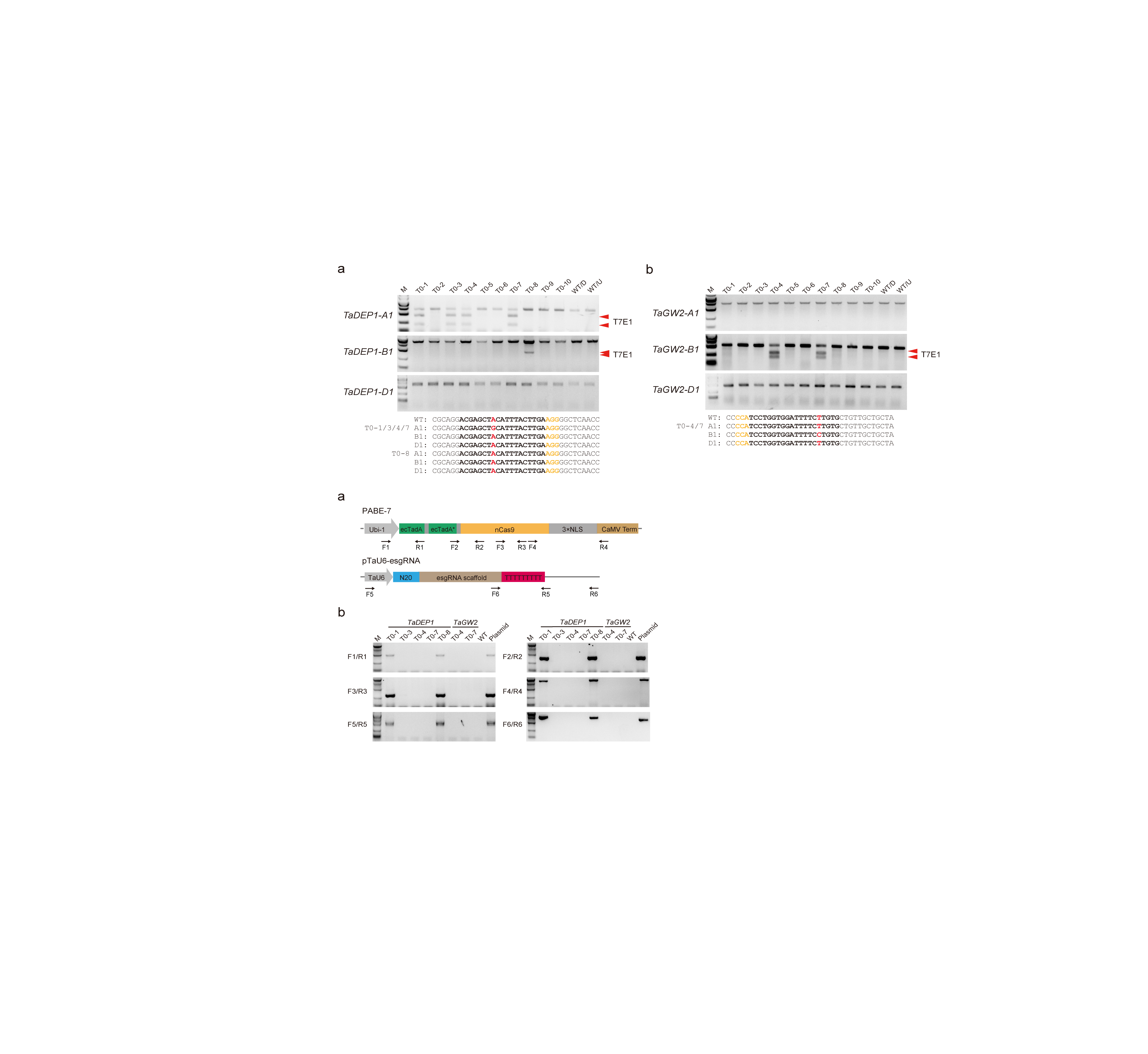


**Figure S7.** Constructs used for base editing of *TaDEP1* and *TaGW2* and detection of transgene integration in the resultant T0 mutants. **(a)** Diagram of the PABE-7 and pTaU6-esgRNA vectors used for base editing of *TaDEP1* and *TaGW2*. The positions of the six primer sets (F1/R1, F2/R2, F3/R3, F4/R4, F5/R5 and F6/R6) used for detecting transgene integration are shown. **(b)** Outcome of the tests for transgene integration using six primer sets for seven mutant plants. None of the six primer sets yielded the expected PCR amplicon in three mutants for *TaDEP1* (T0-3, T0-4 and T0-7) and two mutants for *TaGW2* (T0-4 and T0-7), indicating that they were transgene free. The negative control for the tests was performed using the genomic DNA extracted from wild type wheat plants (cv Kenong 199). The positive control for the tests was conducted with the plasmid DNA of PABE-7 or pTaU6-esgRNA, with the anticipated products being indicated by red arrowheads.

**Table S1.** Description of sgRNA target sites and sequences.

| Gene name | Target sequence | Oligo (5'-3') | Application |
| --- | --- | --- | --- |
| *mGFP* | **CCT**TCACCTACGGCGTGTAGTGC | F:GGCGGCACTACACGCCGTAGGTGA  R:AAACTCACCTACGGCGTGTAGTGC | Constructing pOsU3-sgRNA |
| *OsALS*-T1 | CCCAAGTGGGGGCGCATTCA**AGG** | F:GGCGCCCAAGTGGGGGCGCATTCA  R:AAACTGAATGCGCCCCCACTTGGG | Constructing pOsU3-sgRNA/esgRNA |
|  |  | F:TGCACCCAAGTGGGGGCGCATTCA  R:AAACTGAATGCGCCCCCACTTGGG | Constructing pOsU3-tRNA-sgRNA |
| *OsALS*-T2 | CCTCATGAACATTCAGGAGC**TGG** | F:GGCGCCTCATGAACATTCAGGAGC  R:AAACGCTCCTGAATGTTCATGAGG | Constructing pOsU3-sgRNA/esgRNA |
|  |  | F:TGCACCTCATGAACATTCAGGAGC  R:AAACGCTCCTGAATGTTCATGAGG | Constructing pOsU3-tRNA-sgRNA |
| *OsCDC48-*T1 | GCTAGCTTTGACATAATCTC**CGG** | F:GGCGGCTAGCTTTGACATAATCTC  R:AAACGAGATTATGTCAAAGCTAGC | Constructing pOsU3-sgRNA |
| *OsCDC48-*T2 | CCAATGCATCCGTGAGAAGA**TGG** | F:GGCGCCAATGCATCCGTGAGAAGA  R:AAACTCTTCTCACGGATGCATTGG | Constructing pOsU3-sgRNA |
| *OsCDC48*-T3 | TAGCACCCATGACAATGACA**TGG** | F:GGCGTAGCACCCATGACAATGACA  R:AAACTGTCATTGTCATGGGTGCTA | Constructing pOsU3-sgRNA/esgRNA |
|  |  | F:TGCATAGCACCCATGACAATGACA  R:AAACTGTCATTGTCATGGGTGCTA | Constructing pOsU3-tRNA-sgRNA |
| *OsAAT* | CAAGGATCCCAGCCCCGTGA**AGG** | F:GGCGCAAGGATCCCAGCCCCGTGA  R:AAACTCACGGGGCTGGGATCCTTG | Constructing pOsU3-sgRNA/esgRNA |
|  |  | F:TGCACAAGGATCCCAGCCCCGTGA  R:AAACTCACGGGGCTGGGATCCTTG | Constructing pOsU3-tRNA-sgRNA |
| *OsDEP1*-T1 | AGCACATGAGAGAACAATAT**TGG** | F:GGCGAGCACATGAGAGAACAATAT  R:AAACATATTGTTCTCTCATGTGCT | Constructing pOsU3-sgRNA/esgRNA |
|  |  | F:TGCAAGCACATGAGAGAACAATAT  R:AAACATATTGTTCTCTCATGTGCT | Constructing pOsU3-tRNA-sgRNA |
| *OsDEP1*-T2 | AGACAAGCTTGGCCCTCTTT**GGG** | F:GGCGAGACAAGCTTGGCCCTCTTT  R:AAACAAAGAGGGCCAAGCTTGTCT | Constructing pOsU3-sgRNA/esgRNA |
|  |  | F:TGCAAGACAAGCTTGGCCCTCTTT  R:AAACAAAGAGGGCCAAGCTTGTCT | Constructing pOsU3-tRNA-sgRNA |
| *OsDEP1*-T3 | ATTTCAAATGGATCTAAACA**GGG** | F:GGCGATTTCAAATGGATCTAAACA  R:AAACTGTTTAGATCCATTTGAAAT | Constructing pOsU3-sgRNA |
| *OsDEP1*-T4 | ACAGATCTTGCCGTCTTTTT**CGG** | F:GGCGACAGATCTTGCCGTCTTTTT  R:AAACAAAAAGACGGCAAGATCTGT | Constructing pOsU3-sgRNA |
| *OsACC-*T1 | **CCC**AGACCGCATTGAGTGCTATG | F:GGCGCATAGCACTCAATGCGGTCT  R:AAACAGACCGCATTGAGTGCTATG | Constructing pOsU3-sgRNA/esgRNA |
|  |  | F:TGCACATAGCACTCAATGCGGTCT  R:AAACAGACCGCATTGAGTGCTATG | Constructing pOsU3-tRNA-sgRNA |
| *OsACC-*T2 | TACTAGTCACACTTGCACTG**TGG** | F:GGCGTACTAGTCACACTTGCACTG  R:AAACCAGTGCAAGTGTGACTAGTA | Constructing pOsU3-sgRNA |
| *OsNRT1.1B-*T1 | ACTAGATATCTAAACCATTA**AGG** | F:GGCGACTAGATATCTAAACCATTA  R:AAACTAATGGTTTAGATATCTAGT | Constructing pOsU3-sgRNA/esgRNA |
|  |  | F:TGCAACTAGATATCTAAACCATTA  R:AAACTAATGGTTTAGATATCTAGT | Constructing pOsU3-tRNA-sgRNA |
| *OsNRT1.1B-*T2 | GGCCATGGCGCCCGCGGCGG**CGG** | F:GGCGGGCCATGGCGCCCGCGGCGG  R:AAACCCGCCGCGGGCGCCATGGCC | Constructing pOsU3-sgRNA |
| *OsEV* | CACACACACACTAGTACCTC**TGG** | F:GGCGCACACACACACTAGTACCTC  R:AAACGAGGTACTAGTGTGTGTGTG | Constructing pOsU3-sgRNA/esgRNA |
|  |  | F:TGCACACACACACACTAGTACCTC  R:AAACGAGGTACTAGTGTGTGTGTG | Constructing pOsU3-tRNA-sgRNA |
| *OsOD* | ACACACACACTAGTACCTCT**GGG** | F:GGCGACACACACACTAGTACCTCT  R:AAACAGAGGTACTAGTGTGTGTGT | Constructing pOsU3-sgRNA/esgRNA |
|  |  | F:TGCAACACACACACTAGTACCTCT  R:AAACAGAGGTACTAGTGTGTGTGT | Constructing pOsU3-tRNA-sgRNA |
| *TaDEP1* | ACGAGCTACATTTACTTGAA**GGG** | F:CTTGACGAGCTACATTTACTTGAA  R:AAACTTCAAGTAAATGTAGCTCGT | Constructing pTaU6-sgRNA/esgRNA |
|  |  | F:TGCAACGAGCTACATTTACTTGAA  R:AAACTTCAAGTAAATGTAGCTCGT | Constructing pTaU6-tRNA-sgRNA |
| *TaEPSPS* | GAGGAAGTAAAGCTCTTCTT**GGG** | F:CTTGGAGGAAGTAAAGCTCTTCTT  R:AAACAAGAAGAGCTTTACTTCCTC | Constructing pTaU6-sgRNA/esgRNA |
|  |  | F:TGCAGAGGAAGTAAAGCTCTTCTT  R:AAACAAGAAGAGCTTTACTTCCTC | Constructing pTaU6-tRNA-sgRNA |
| *TaGW2* | CACAAGAAAATCCACCAGGA**TGG** | F:CTTGCACAAGAAAATCCACCAGGA  R:AAACTCCTGGTGGATTTTCTTGTG | Constructing pTaU6-sgRNA/esgRNA |
|  |  | F:TGCACACAAGAAAATCCACCAGGA  R:AAACTCCTGGTGGATTTTCTTGTG | Constructing pTaU6-tRNA-sgRNA |

The A/T bases marked in red were found to be edited by plant ABEs. The PAM motif in each target sequence is shown in bold.

**Table S2. Potential off-target sites analyzed for *OsACC*-T1 endogenous genomic loci.**

| Potential off-taget site | Sequence^a^ | No. of mismatches | Target gene loci | Mutation genotype | Detection Method |
| --- | --- | --- | --- | --- | --- |
| On-target | **CCC**AGACCGCATTGAGTGCTATG | 0 | LOC_Os05g22940 | A to G | T7E1/Sanger Sequencing |
| Off-target-1 | CATAGCACTCAActCaGTtT**GGG** | 4 | LOC_Os08g35020 | Neither A to G conversion nor indels were found | T7E1/Sanger Sequencing |
| Off-target-2 | aATAGCACTCAtTGaGaTCT**TGG** | 4 | LOC_Os05g30010 |  | T7E1/Sanger Sequencing |
| Off-target-3 | CATAGCACTtAATGtGGgCg**GAG** | 4 | LOC_Os03g46820 |  | T7E1/Sanger Sequencing |

^a^ The mismatch bases in the fourteen potential off-target sites are shown as lower case letters. The PAM motif is written in bold.

**Table S3.** PCR primers used in this study.

| Primer name | Primer sequence (5'-3') | Application |
| --- | --- | --- |
| mGFP-F  mGFP-R | TTCACCTACGGCGTGTAGTGCTTCAGCCGCT  AGCGGCTGAAGCACTACACGCCGTAGGTGAA | Constructing the vector pJIT163-Ubi-mGFP |
| OsALS-T1-F  OsALS-T1-R | CTGTCTTCGGCTGGTCTGGG TGCCAAGCACATCAAACAAGTAAA | Amplifying the *OsALS* target site 1 and 1^st^ PCR for deep sequencing |
| OsALS-T1-A-F  OsALS-T1-A-R | CGATGTCCGCCATCAAGAAGATGCTC  TGACCACTGGTGCTTTGCCAACATAC | 2^nd^ PCR for deep sequencing of *OsALS*-T1 in rice protoplasts treated by PABE-2 (Fig. 1e) or PABE-7 (Fig. 2a) with the native sgRNA |
| OsALS-T1-B-F  OsALS-T1-B-R | ACAGTGCCGCCATCAAGAAGATGCTC  GCCAATCTGGTGCTTTGCCAACATAC | 2^nd^ PCR for deep sequencing of *OsALS*-T1 in rice protoplasts treated by PABE-7 with the sgRNA (Fig. 1e) or esgRNA (Fig. 2a) |
| OsALS-T1-C-F  OsALS-T1-C-R | CAGATCCCGCCATCAAGAAGATGCTC  CTTGTACTGGTGCTTTGCCAACATAC | 2^nd^ PCR for deep sequencing of *OsALS*-T1 in control rice protoplasts (Fig. 1e) or treated by PABE-7 with the tRNA-sgRNA (Fig. 2a) |
| OsALS-T1-D-F  OsALS-T1-D-R | AGTCAACCGCCATCAAGAAGATGCTC  AGTTCCCTGGTGCTTTGCCAACATAC | 2^nd^ PCR for deep sequencing of *OsALS*-T1 in rice protoplasts treated by wild type Cas9 with the native sgRNA (Fig. 2c) |
| OsALS-T1-E-F  OsALS-T1-E-R | GTAGAGCCGCCATCAAGAAGATGCTC  GTCCGCCTGGTGCTTTGCCAACATAC | 2^nd^ PCR for deep sequencing of *OsALS*-T1 in rice protoplasts treated by wild type Cas9 with the esgRNA (Fig. 2c) |
| OsALS-T1-F-F  OsALS-T1-F-R | GTTTCGCCGCCATCAAGAAGATGCTC  CGTACGCTGGTGCTTTGCCAACATAC | 2^nd^ PCR for deep sequencing of *OsALS*-T1 in rice protoplasts treated by wild type Cas9 with the tRNA-sgRNA (Fig. 2c) |
| OsALS-T1-G-F  OsALS-T1-G-R | ACTGATCCGCCATCAAGAAGATGCTC  ATGAGCCTGGTGCTTTGCCAACATAC | 2^nd^ PCR for deep sequencing of *OsALS*-T1 in control rice protoplasts (Fig. 2a and Fig. 2c) |
| OsALS-T2-F  OsALS-T2-R | GCATGGCACAATGAGTTGGACC  GTCATTCAGGTCAAACATAGGCC | Amplifying the *OsALS* target site 2 and 1^st^ PCR for deep sequencing |
| OsALS-T2-A-F  OsALS-T2-A-R | CGATGTTGGTGCTTCTGTGGCTAAC  TGACCACCATTGCACCACCATACCC | 2^nd^ PCR for deep sequencing of *OsALS*-T2 in rice protoplasts treated by PABE-2 (Fig. 1e) or PABE-7 (Fig. 2a) with the native sgRNA |
| OsALS-T2-B-F  OsALS-T2-B-R | ACAGTGTGGTGCTTCTGTGGCTAAC  GCCAATCCATTGCACCACCATACCC | 2^nd^ PCR for deep sequencing of *OsALS*-T2 in rice protoplasts treated by PABE-7 with the sgRNA (Fig. 1e) or esgRNA (Fig. 2a) |
| OsALS-T2-C-F  OsALS-T2-C-R | CAGATCTGGTGCTTCTGTGGCTAAC  CTTGTACCATTGCACCACCATACCC | 2^nd^ PCR for deep sequencing of *OsALS*-T2 in control rice protoplasts (Fig. 1e) or treated by PABE-7 with the tRNA-sgRNA (Fig. 2a) |
| OsALS-T2-D-F  OsALS-T2-D-R | AGTCAATGGTGCTTCTGTGGCTAAC  AGTTCCCCATTGCACCACCATACCC | 2^nd^ PCR for deep sequencing of *OsALS*-T2 in rice protoplasts treated by wild type Cas9 with the native sgRNA (Fig. 2c) |
| OsALS-T2-E-F  OsALS-T2-E-R | GTAGAGTGGTGCTTCTGTGGCTAAC  GTCCGCCCATTGCACCACCATACCC | 2^nd^ PCR for deep sequencing of *OsALS*-T2 in rice protoplasts treated by wild type Cas9 with the esgRNA (Fig. 2c) |
| OsALS-T2-F-F  OsALS-T2-F-R | GTTTCGTGGTGCTTCTGTGGCTAAC  CGTACGCCATTGCACCACCATACCC | 2^nd^ PCR for deep sequencing of *OsALS*-T2 in rice protoplasts treated by wild type Cas9 with the tRNA-sgRNA (Fig. 2c) |
| OsALS-T2-G-F  OsALS-T2-G-R | ACTGATTGGTGCTTCTGTGGCTAAC  ATGAGCCCATTGCACCACCATACCC | 2^nd^ PCR for deep sequencing of *OsALS*-T2 in control rice protoplasts (Fig. 2a and Fig. 2c) |
| OsCDC48-T1-F  OsCDC48-T1-R | CAAGTCTATTGGTGTGAAGCCTC  TGATAACTCACATCTTCAGCCAGC | Amplifying the *OsCDC48* target site 1 and 1^st^ PCR for deep sequencing |
| OsCDC48-T1-A-F  OsCDC48-T1-A-R | CGATGTACCCTCATTGCTAGAGCTG  TGACCAGCTATGGAGTCTATCTCATCGATG | 2^nd^ PCR for deep sequencing of *OsCDC48*-T1 in rice protoplasts treated by PABE-2 with the native sgRNA (Fig. 1e) |
| OsCDC48-T1-B-F  OsCDC48-T1-B-R | ACAGTGACCCTCATTGCTAGAGCTG  GCCAATGCTATGGAGTCTATCTCATCGATG | 2^nd^ PCR for deep sequencing of *OsCDC48*-T1 in rice protoplasts treated by PABE-7 with the sgRNA (Fig. 1e) |
| OsCDC48-T1-C-F  OsCDC48-T1-C-R | CAGATCACCCTCATTGCTAGAGCTG  CTTGTAGCTATGGAGTCTATCTCATCGATG | 2^nd^ PCR for deep sequencing of *OsCDC48*-T1 in control rice protoplasts (Fig. 1e) |
| OsCDC48-T2-F  OsCDC48-T2-R | GCTTGAAGTTCTTCGGATTCACACC  AATAAGTACCTCCTGCAGCTCCC | Amplifying the *OsCDC48* target site 2 and 1^st^ PCR for deep sequencing |
| OsCDC48-T2-A-F  OsCDC48-T2-A-R | CGATGTGACACTCATGGGTATGTGGG  TGACCACCTAGTGCAGTCTTGAAGTGATC | 2^nd^ PCR for deep sequencing of *OsCDC48*-T2 in rice protoplasts treated by PABE-2 with the native sgRNA (Fig. 1e) |
| OsCDC48-T2-B-F  OsCDC48-T2-B-R | ACAGTGGACACTCATGGGTATGTGGG  GCCAATCCTAGTGCAGTCTTGAAGTGATC | 2^nd^ PCR for deep sequencing of *OsCDC48*-T2 in rice protoplasts treated by PABE-7 with the sgRNA (Fig. 1e) |
| OsCDC48-T2-C-F  OsCDC48-T2-C-R | CAGATCGACACTCATGGGTATGTGGG  CTTGTACCTAGTGCAGTCTTGAAGTGATC | 2^nd^ PCR for deep sequencing of *OsCDC48*-T2 in control rice protoplasts (Fig. 1e) |
| OsCDC48-T3-F  OsCDC48-T3-R | TCTTTCTGATTAATGGCCCGGAG  GGATGCATTGGAGAGCAGCC | Amplifying the *OsCDC48* target site and 1^st^ PCR for deep sequencing |
| OsCDC48-T3-A-F  OsCDC48-T3-A-R | CGATGTAAGTTGAGAGGCGCATCG  TGACCACATCAGGAACACCAATGTCAATC | 2^nd^ PCR for deep sequencing of *OsCDC48*-T3 in rice protoplasts treated by PABE-2 (Fig. 1e) or PABE-7 (Fig. 2a) with the native sgRNA |
| OsCDC48-T3-B-F  OsCDC48-T3-B-R | ACAGTGAAGTTGAGAGGCGCATCG  GCCAATCATCAGGAACACCAATGTCAATC | 2^nd^ PCR for deep sequencing of *OsCDC48*-T3 in rice protoplasts treated by PABE-7 with the sgRNA (Fig. 1e) or esgRNA (Fig. 2a) |
| OsCDC48-T3-C-F  OsCDC48-T3-C-R | CAGATCAAGTTGAGAGGCGCATCG  CTTGTACATCAGGAACACCAATGTCAATC | 2^nd^ PCR for deep sequencing of *OsCDC48*-T3 in control rice protoplasts (Fig. 1e) or treated by PABE-7 with the tRNA-sgRNA (Fig. 2a) |
| OsCDC48-T3-D-F  OsCDC48-T3-D-R | AGTCAAAAGTTGAGAGGCGCATCG  AGTTCCCATCAGGAACACCAATGTCAATC | 2^nd^ PCR for deep sequencing of *OsCDC48*-T3 in rice protoplasts treated by wild type Cas9 with the native sgRNA (Fig. 2c) |
| OsCDC48-T3-E-F  OsCDC48-T3-E-R | GTAGAGAAGTTGAGAGGCGCATCG  GTCCGCCATCAGGAACACCAATGTCAATC | 2^nd^ PCR for deep sequencing of *OsCDC48*-T3 in rice protoplasts treated by wild type Cas9 with the esgRNA (Fig. 2c) |
| OsCDC48-T3-F-F  OsCDC48-T3-F-R | GTTTCGAAGTTGAGAGGCGCATCG  CGTACGCATCAGGAACACCAATGTCAATC | 2^nd^ PCR for deep sequencing of *OsCDC48*-T3 in rice protoplasts treated by wild type Cas9 with the tRNA-sgRNA (Fig. 2c) |
| OsCDC48-T3-G-F  OsCDC48-T3-G-R | ACTGATAAGTTGAGAGGCGCATCG  ATGAGCCATCAGGAACACCAATGTCAATC | 2^nd^ PCR for deep sequencing of *OsCDC48*-T3 in control rice protoplasts (Fig. 2a and Fig. 2c) |
| OsAAT-F  OsAAT-R | AGGTTAAGTACGCTGGTGCGC  GACGATTCAAAGCAAGAATGGTGCC | Amplifying the *OsAAT* target site and 1^st^ PCR for deep sequencing |
| OsAAT-A-F  OsAAT-A-R | CGATGTTCGACCTGATCGGTGCTC  TGACCAATCCACCACCAATCCAATCC | 2^nd^ PCR for deep sequencing of *OsAAT* in rice protoplasts treated by PABE-2 (Fig. 1e) or PABE-7 (Fig. 2a) with the native sgRNA |
| OsAAT-B-F  OsAAT-B-R | ACAGTGTCGACCTGATCGGTGCTC  GCCAATATCCACCACCAATCCAATCC | 2^nd^ PCR for deep sequencing of *OsAAT* in rice protoplasts treated by PABE-7 with the sgRNA (Fig. 1e) or esgRNA (Fig. 2a) |
| OsAAT-C-F  OsAAT-C-R | CAGATCTCGACCTGATCGGTGCTC  CTTGTAATCCACCACCAATCCAATCC | 2^nd^ PCR for deep sequencing of *OsAAT* in control rice protoplasts (Fig. 1e) or treated by PABE-7 with the tRNA-sgRNA (Fig. 2a) |
| OsAAT-D-F  OsAAT-D-R | AGTCAATCGACCTGATCGGTGCTC  AGTTCCATCCACCACCAATCCAATCC | 2^nd^ PCR for deep sequencing of *OsAAT* in rice protoplasts treated by wild type Cas9 with the native sgRNA (Fig. 2c) |
| OsAAT-E-F  OsAAT-E-R | GTAGAGTCGACCTGATCGGTGCTC  GTCCGCATCCACCACCAATCCAATCC | 2^nd^ PCR for deep sequencing of *OsAAT* in rice protoplasts treated by wild type Cas9 with the esgRNA (Fig. 2c) |
| OsAAT-F-F  OsAAT-F-R | GTTTCGTCGACCTGATCGGTGCTC  CGTACGATCCACCACCAATCCAATCC | 2^nd^ PCR for deep sequencing of *OsAAT* in rice protoplasts treated by wild type Cas9 with the tRNA-sgRNA (Fig. 2c) |
| OsAAT-G-F  OsAAT-G-R | ACTGATTCGACCTGATCGGTGCTC  ATGAGCATCCACCACCAATCCAATCC | 2^nd^ PCR for deep sequencing of *OsAAT* in control rice protoplasts (Fig. 2a and Fig. 2c) |
| OsDEP1-T1-F  OsDEP1-T1-R | TCAGCCTGCAGTACTGAATTATC  GGGCCTAAGTGTGACATACAAG | Amplifying the *OsDEP1* target site 1 and 1^st^ PCR for deep sequencing |
| OsDEP1- T1-A-F  OsDEP1- T1-A-R | CGATGTACAGGTAGAAAAACTTTTGTGGG  TGACCACATGAAGGGCAGTAGTACATACTC | 2^nd^ PCR for deep sequencing of *OsDEP1*-T1 in rice protoplasts treated by PABE-2 (Fig. 1e) or PABE-7 (Fig. 2a) with the native sgRNA |
| OsDEP1- T1-B-F  OsDEP1- T1-B-R | ACAGTGACAGGTAGAAAAACTTTTGTGGG  GCCAATCATGAAGGGCAGTAGTACATACTC | 2^nd^ PCR for deep sequencing of *OsDEP1*-T1 in rice protoplasts treated by PABE-7 with the sgRNA (Fig. 1e) or esgRNA (Fig. 2a) |
| OsDEP1- T1-C-F  OsDEP1- T1-C-R | CAGATCACAGGTAGAAAAACTTTTGTGGG  CTTGTACATGAAGGGCAGTAGTACATACTC | 2^nd^ PCR for deep sequencing of *OsDEP1*-T1 in control rice protoplasts (Fig. 1e) or treated by PABE-7 with the tRNA-sgRNA (Fig. 2a) |
| OsDEP1- T1-D-F  OsDEP1- T1-D-R | AGTCAAACAGGTAGAAAAACTTTTGTGGG  AGTTCCCATGAAGGGCAGTAGTACATACTC | 2^nd^ PCR for deep sequencing of *OsDEP1*-T1 in rice protoplasts treated by wild type Cas9 with the native sgRNA (Fig. 2c) |
| OsDEP1- T1-E-F  OsDEP1- T1-E-R | GTAGAGACAGGTAGAAAAACTTTTGTGGG  GTCCGCCATGAAGGGCAGTAGTACATACTC | 2^nd^ PCR for deep sequencing of *OsDEP1*-T1 in rice protoplasts treated by wild type Cas9 with the esgRNA (Fig. 2c) |
| OsDEP1- T1-F-F  OsDEP1- T1-F-R | GTTTCGACAGGTAGAAAAACTTTTGTGGG  CGTACGCATGAAGGGCAGTAGTACATACTC | 2^nd^ PCR for deep sequencing of *OsDEP1*-T1 in rice protoplasts treated by wild type Cas9 with the tRNA-sgRNA (Fig. 2c) |
| OsDEP1- T1-G-F  OsDEP1- T1-G-R | ACTGATACAGGTAGAAAAACTTTTGTGGG  ATGAGCCATGAAGGGCAGTAGTACATACTC | 2^nd^ PCR for deep sequencing of *OsDEP1*-T1 in control rice protoplasts (Fig. 2a and Fig. 2c) |
| OsDEP1-T2-F  OsDEP1-T2-R | GCAAGTAGGATGCTGTGAAG  GAAGTTCTCTGATATCTGAAGAGCTTCTG | Amplifying the *OsDEP1* target site 2 and 1^st^ PCR for deep sequencing |
| OsDEP1-T2-A-F  OsDEP1-T2-A-R | CGATGTCTTGTAGTACTGTTTCTAGGCGG  TGACCAAGGTCAGTTGTCCATGCCCA | 2^nd^ PCR for deep sequencing of *OsDEP1*-T2 in rice protoplasts treated by PABE-2 (Fig. 1e) or PABE-7 (Fig. 2a) with the native sgRNA |
| OsDEP1-T2-B-F  OsDEP1-T2-B-R | ACAGTGCTTGTAGTACTGTTTCTAGGCGG  GCCAATAGGTCAGTTGTCCATGCCCA | 2^nd^ PCR for deep sequencing of *OsDEP1*-T2 in rice protoplasts treated by PABE-7 with the sgRNA (Fig. 1e) or esgRNA (Fig. 2a) |
| OsDEP1-T2-C-F  OsDEP1-T2-C-R | CAGATCCTTGTAGTACTGTTTCTAGGCGG  CTTGTAAGGTCAGTTGTCCATGCCCA | 2^nd^ PCR for deep sequencing of *OsDEP1*-T2 in control rice protoplasts (Fig. 1e) or treated by PABE-7 with the tRNA-sgRNA (Fig. 2a) |
| OsDEP1-T2-D-F  OsDEP1-T2-D-R | AGTCAAGGTAGCGTACTGTTTCTAGGCGG  AGTTCCAGGTCAGTTGTCCATGCCCA | 2^nd^ PCR for deep sequencing of *OsDEP1*-T2 in rice protoplasts treated by wild type Cas9 with the native sgRNA (Fig. 2c) |
| OsDEP1-T2-E-F  OsDEP1-T2-E-R | GTAGAGGGTAGCGTACTGTTTCTAGGCGG  GTCCGCAGGTCAGTTGTCCATGCCCA | 2^nd^ PCR for deep sequencing of *OsDEP1*-T2 in rice protoplasts treated by wild type Cas9 with the esgRNA (Fig. 2c) |
| OsDEP1-T2-F-F  OsDEP1-T2-F-R | GTTTCGGGTAGCGTACTGTTTCTAGGCGG  CGTACGAGGTCAGTTGTCCATGCCCA | 2^nd^ PCR for deep sequencing of *OsDEP1*-T2 in rice protoplasts treated by wild type Cas9 with the tRNA-sgRNA (Fig. 2c) |
| OsDEP1-T2-G-F  OsDEP1-T2-G-R | ACTGATCTTGTAGTACTGTTTCTAGGCGG  ATGAGCAGGTCAGTTGTCCATGCCCA | 2^nd^ PCR for deep sequencing of *OsDEP1*-T2 in control rice protoplasts (Fig. 2a and Fig. 2c) |
| OsDEP1-T3-F  OsDEP1-T3-R | CTTGGCAATTTGGCATTTGGCATT  GAAACAAATCTCAGGACTGTGAGC | Amplifying the *OsDEP1*-T3 target site 1 and 1^st^ PCR for deep sequencing |
| OsDEP1-T3-A-F  OsDEP1-T3-A-R | CGATGTACACCAACTAGTACTTTTACAATAC  TGACCATTTCAAACAAGACTTCAATGCTGG | 2^nd^ PCR for deep sequencing of *OsDEP1*-T3 in rice protoplasts treated by PABE-2 with the native sgRNA (Fig. 1e) |
| OsDEP1-T3-B-F  OsDEP1-T3-B-R | ACAGTGACACCAACTAGTACTTTTACAATAC  GCCAATTTTCAAACAAGACTTCAATGCTGG | 2^nd^ PCR for deep sequencing of *OsDEP1*-T3 in rice protoplasts treated by PABE-7 with the sgRNA (Fig. 1e) |
| OsDEP1-T3-C-F  OsDEP1-T3-C-R | CAGATCACACCAACTAGTACTTTTACAATAC  CTTGTATTTCAAACAAGACTTCAATGCTGG | 2^nd^ PCR for deep sequencing of *OsDEP1*-T3 in control rice protoplasts (Fig. 1e) |
| OsDEP1-T4-F  OsDEP1-T4-R | ACATGACCCACTAATACCAACG  TTGGCAGTAGTACAGATTATTATGCC | Amplifying the *OsDEP1*-T4 target site 1 and 1^st^ PCR for deep sequencing |
| OsDEP1-T4-A-F  OsDEP1-T4-A-R | CGATGTCTTGTAGTACTGTTTCTAGGCGG  TGACCAAGGTCAGTTGTCCATGCCCA | 2^nd^ PCR for deep sequencing of *OsDEP1*-T4 in rice protoplasts treated by PABE-2 with the native sgRNA (Fig. 1e) |
| OsDEP1-T4-B-F  OsDEP1-T4-B-R | ACAGTGCTTGTAGTACTGTTTCTAGGCGG  GCCAATAGGTCAGTTGTCCATGCCCA | 2^nd^ PCR for deep sequencing of *OsDEP1*-T4 in rice protoplasts treated by PABE-7 with the sgRNA (Fig. 1e) |
| OsDEP1-T4-C-F  OsDEP1-T4-C-R | CAGATCCTTGTAGTACTGTTTCTAGGCGG  CTTGTAAGGTCAGTTGTCCATGCCCA | 2^nd^ PCR for deep sequencing of *OsDEP1*-T4 in control rice protoplasts (Fig. 1e) |
| OsACC-T1-F  OsACC-T1-R | CATGATGCAAACTATCCCTGCTGAC  GAAAGATCGTGATTCTTCCCAGTCCA | Amplifying the *OsACC*-T1 target site and 1^st^ PCR for deep sequencing |
| OsACC-T1-A-F  OsACC-T1-A-R | CGATGTTTCCCATGGCTGCAGAGC  TGACCAATCCTGGAGTTCCTCTGACC | 2^nd^ PCR for deep sequencing of *OsACC*-T1 in rice protoplasts treated by PABE-2 (Fig. 1e) or PABE-7 (Fig. 2a) with the native sgRNA |
| OsACC-T1-B-F  OsACC-T1-B-R | ACAGTGTTCCCATGGCTGCAGAGC  GCCAATATCCTGGAGTTCCTCTGACC | 2^nd^ PCR for deep sequencing of *OsACC*-T1 in rice protoplasts treated by PABE-7 with the sgRNA (Fig. 1e) or esgRNA (Fig. 2a) |
| OsACC-T1-C-F  OsACC-T1-C-R | CAGATCTTCCCATGGCTGCAGAGC  CTTGTAATCCTGGAGTTCCTCTGACC | 2^nd^ PCR for deep sequencing of *OsACC*-T1 in control rice protoplasts (Fig. 1e) or treated by PABE-7 with the tRNA-sgRNA (Fig. 2a) |
| OsACC-T1-D-F  OsACC-T1-D-R | AGTCAATTCCCATGGCTGCAGAGC  AGTTCCATCCTGGAGTTCCTCTGACC | 2^nd^ PCR for deep sequencing of *OsACC*-T1 in rice protoplasts treated by wild type Cas9 with the native sgRNA (Fig. 2c) |
| OsACC-T1-E-F  OsACC-T1-E-R | GTAGAGTTCCCATGGCTGCAGAGC  GTCCGCATCCTGGAGTTCCTCTGACC | 2^nd^ PCR for deep sequencing of *OsACC*-T1 in rice protoplasts treated by wild type Cas9 with the esgRNA (Fig. 2c) |
| OsACC-T1-F-F  OsACC-T1-F-R | GTTTCGTTCCCATGGCTGCAGAGC  CGTACGATCCTGGAGTTCCTCTGACC | 2^nd^ PCR for deep sequencing of *OsACC*-T1 in rice protoplasts treated by wild type Cas9 with the tRNA-sgRNA (Fig. 2c) |
| OsACC-T1-G-F  OsACC-T1-G-R | ACTGATTTCCCATGGCTGCAGAGC  ATGAGCATCCTGGAGTTCCTCTGACC | 2^nd^ PCR for deep sequencing of *OsACC*-T1 in control rice protoplasts (Fig. 2a and Fig. 2c) |
| OsACC-T2-F  OsACC-T2-R | AGCTTCTTGATCTTGTTCCAGTTTCAG  CCTCACTGCAGTTTCAAATGCCTACA | Amplifying the *OsACC*-T2 target site 1 and 1^st^ PCR for deep sequencing |
| OsACC-T2-A-F  OsACC-T2-A-R | CGATGTTGAAACTTAAGTTGGACTGCG  TGACCACTGGGTATGAGGACCAGCC | 2^nd^ PCR for deep sequencing of *OsACC*-T2 in rice protoplasts treated by PABE-2 with the native sgRNA (Fig. 1e) |
| OsACC-T2-B-F  OsACC-T2-B-R | ACAGTGTGAAACTTAAGTTGGACTGCG  GCCAATCTGGGTATGAGGACCAGCC | 2^nd^ PCR for deep sequencing of *OsACC*-T2 in rice protoplasts treated by PABE-7 with the sgRNA (Fig. 1e) |
| OsACC-T2-C-F  OsACC-T2-C-R | CAGATCTGAAACTTAAGTTGGACTGCG  CTTGTACTGGGTATGAGGACCAGCC | 2^nd^ PCR for deep sequencing of *OsACC*-T2 in control rice protoplasts (Fig. 1e) |
| OsNRT1.1B-T1-F  OsNRT1.1B-T1-R | AGCTAGGAGTAGAGAACGAGACATATAC  GTTGGGAGAATAGCTGAAGCTATCGG | Amplifying the *OsNRT1.1B*-T1 target site and 1^st^ PCR for deep sequencing |
| OsNRT1.1B-T1-A-F  OsNRT1.1B-T1-A-R | CGATGTACTCCAGCCACTCACTGTC  TGACCAGTCGTTGGGACTGGGCTAC | 2^nd^ PCR for deep sequencing of *OsNRT1.1B*-T1 in rice protoplasts treated by PABE-2 (Fig. 1e) or PABE-7 (Fig. 2a) with the native sgRNA |
| OsNRT1.1B-T1-B-F  OsNRT1.1B-T1-B-R | ACAGTGACTCCAGCCACTCACTGTC  GCCAATGTCGTTGGGACTGGGCTAC | 2^nd^ PCR for deep sequencing of *OsNRT1.1B*-T1 in rice protoplasts treated by PABE-7 with the sgRNA (Fig. 1e) or esgRNA (Fig. 2a) |
| OsNRT1.1B-T1-C-F  OsNRT1.1B-T1-C-R | CAGATCACTCCAGCCACTCACTGTC  CTTGTAGTCGTTGGGACTGGGCTAC | 2^nd^ PCR for deep sequencing of *OsNRT1.1B*-T1 in control rice protoplasts (Fig. 1e) or treated by PABE-7 with the tRNA-sgRNA (Fig. 2a) |
| OsNRT1.1B-T1-D-F  OsNRT1.1B-T1-D-R | AGTCAAACTCCAGCCACTCACTGTC  AGTTCCGTCGTTGGGACTGGGCTAC | 2^nd^ PCR for deep sequencing of *OsNRT1.1B*-T1 in rice protoplasts treated by wild type Cas9 with the native sgRNA (Fig. 2c) |
| OsNRT1.1B-T1-E-F  OsNRT1.1B-T1-E-R | GTAGAGACTCCAGCCACTCACTGTC  GTCCGCGTCGTTGGGACTGGGCTAC | 2^nd^ PCR for deep sequencing of *OsNRT1.1B*-T1 in rice protoplasts treated by wild type Cas9 with the esgRNA (Fig. 2c) |
| OsNRT1.1B-T1-F-F  OsNRT1.1B-T1-F-R | GTTTCGACTCCAGCCACTCACTGTC  CGTACGGTCGTTGGGACTGGGCTAC | 2^nd^ PCR for deep sequencing of *OsNRT1.1B*-T1 in rice protoplasts treated by wild type Cas9 with the tRNA-sgRNA (Fig. 2c) |
| OsNRT1.1B-T1-G-F  OsNRT1.1B-T1-G-R | ACTGATACTCCAGCCACTCACTGTC  ATGAGCGTCGTTGGGACTGGGCTAC | 2^nd^ PCR for deep sequencing of *OsNRT1.1B*-T1 in control rice protoplasts (Fig. 2a and Fig. 2c) |
| OsNRT1.1B-T2-F  OsNRT1.1B-T2-R | TCTTCGTGGGCCTCTCCCTCTC  ATACGTGCGATGAGAATACACCCAA | Amplifying the *OsNRT1.1B*-T2 target site 1 and 1^st^ PCR for deep sequencing |
| OsNRT1.1B-T2-A-F  OsNRT1.1B-T2-A-R | CGATGTGCACCTGCGCCATCGGC  TGACCAGGTTGATGGCGCTGATGACG | 2^nd^ PCR for deep sequencing of *OsNRT1.1B*-T2 in rice protoplasts treated by PABE-2 with the native sgRNA (Fig. 1e) |
| OsNRT1.1B-T2-B-F  OsNRT1.1B-T2-B-R | ACAGTGGCACCTGCGCCATCGGC  GCCAATGGTTGATGGCGCTGATGACG | 2^nd^ PCR for deep sequencing of *OsNRT1.1B*-T2 in rice protoplasts treated by PABE-7 with the sgRNA (Fig. 1e) |
| OsNRT1.1B-T2-C-F  OsNRT1.1B-T2-C-R | CAGATCGCACCTGCGCCATCGGC  CTTGTAGGTTGATGGCGCTGATGACG | 2^nd^ PCR for deep sequencing of *OsNRT1.1B*-T2 in control rice protoplasts (Fig. 1e) |
| OsEV-F  OsEV-R | GGGAGATGAGAGAGCTTGTGCC  GAGTAGTGTAGTACTGAAGAAGCACAGC | Amplifying the *OsEV* target site and 1^st^ PCR for deep sequencing |
| OsEV-A-F  OsEV-A-R | ATCACGCCTTGCTTTCATTCTTCAGTGC  TTAGGCGCAGACCAAGATCCCAAGAAC | 2^nd^ PCR for deep sequencing of *OsEV* in rice protoplasts treated by PABE-7 with the native sgRNA (Fig. 1e and Fig. 2a), 14nt esgRNA (Fig. 2c), wild type Cas9 with the 14nt esgRNA or control rice protoplasts (Fig. 2c ; Additional file 1: Figure S4) |
| OsEV-B-F  OsEV-B-R | ACTTGACCTTGCTTTCATTCTTCAGTGC  GATCAGGCAGACCAAGATCCCAAGAAC | 2^nd^ PCR for deep sequencing of *OsEV* in rice protoplasts treated by PABE-2 with sgRNA (Fig. 1e), PABE-7 with the esgRNA (Fig. 2a), 15nt esgRNA (Fig. 2c), wild type Cas9 with the 15nt esgRNA or control rice protoplasts (Fig. 2c ; Additional file 1: Figure S4) |
| OsEV-C-F  OsEV-C-R | TAGCTTCCTTGCTTTCATTCTTCAGTGC  GGCTACGCAGACCAAGATCCCAAGAAC | 2^nd^ PCR for deep sequencing of *OsEV* in control rice protoplasts (Fig. 1e), or rice protoplasts treated by PABE-7 with the tRNA-sgRNA (Fig. 2a), 16nt esgRNA (Fig. 2c), wild type Cas9 with the 16nt esgRNA or control rice protoplasts (Fig. 2c ; Additional file 1: Figure S4) |
| OsEV-D-F  OsEV-D-R | ATGTCACCTTGCTTTCATTCTTCAGTGC  CCGTCCGCAGACCAAGATCCCAAGAAC | 2^nd^ PCR for deep sequencing of *OsEV* in rice protoplasts treated by wild type Cas9 with the native sgRNA (Fig. 2c), PABE-7 with the 17nt esgRNA (Fig. 2c), wild type Cas9 with the 17nt esgRNA or control rice protoplasts (Fig. 2c ; Additional file 1: Figure S4) |
| OsEV-E-F  OsEV-E-R | GTGAAACCTTGCTTTCATTCTTCAGTGC  GTGGCCGCAGACCAAGATCCCAAGAAC | 2^nd^ PCR for deep sequencing of *OsEV* in rice protoplasts treated by wild type Cas9 with the esgRNA (Fig. 2c), PABE-7 with the 18nt esgRNA (Fig. 2c), wild type Cas9 with the 18nt esgRNA or control rice protoplasts (Fig. 2c ; Additional file 1: Figure S4) |
| OsEV-F-F  OsEV-F-R | GAGTGGCCTTGCTTTCATTCTTCAGTGC  GGTAGCGCAGACCAAGATCCCAAGAAC | 2^nd^ PCR for deep sequencing of *OsEV* in rice protoplasts treated by wild type Cas9 with the tRNA-sgRNA (Fig. 2c), PABE-7 with the 19nt esgRNA (Fig. 2c), wild type Cas9 with the 19nt esgRNA or control rice protoplasts (Fig. 2c ; Additional file 1: Figure S4) |
| OsEV-G-F  OsEV-G-R | ATTCCTCCTTGCTTTCATTCTTCAGTGC  CAAAAGGCAGACCAAGATCCCAAGAAC | 2^nd^ PCR for deep sequencing of *OsEV* in control rice protoplasts (Fig. 2a and Fig. 2c), PABE-7 with the 20nt esgRNA (Fig. 2c), wild type Cas9 with the 20nt esgRNA or control rice protoplasts (Fig. 2c ; Additional file 1: Figure S4) |
| OsEV-H-F  OsEV-H-R | CACGATCCTTGCTTTCATTCTTCAGTGC  CACTCAGCAGACCAAGATCCCAAGAAC | 2^nd^ PCR for deep sequencing of *OsOD* in rice protoplasts treated by wild type Cas9 with the 18nt esgRNA (Additional file 1: Figure S4), *OsEV* in rice protoplasts treated by wild type Cas9 with the 18nt esgRNA (Additional file 1: Figure S4) or control rice protoplasts (Fig. 2c ; Additional file 1: Figure S4) |
| OsEV-I-F  OsEV-I-R | CATTTTCCTTGCTTTCATTCTTCAGTGC  CCAACAGCAGACCAAGATCCCAAGAAC | 2^nd^ PCR for deep sequencing of *OsOD* in rice protoplasts treated by wild type Cas9 with the 19nt esgRNA (Additional file 1: Figure S4), *OsEV* in rice protoplasts treated by wild type Cas9 with the 19nt esgRNA (Additional file 1: Figure S4) or control rice protoplasts (Fig. 2c ; Additional file 1: Figure S4) |
| OsEV-J-F  OsEV-J-R | CTATACCCTTGCTTTCATTCTTCAGTGC  CTCAGAGCAGACCAAGATCCCAAGAAC | 2^nd^ PCR for deep sequencing of *OsOD* in rice protoplasts treated by wild type Cas9 with the 20nt esgRNA (Additional file 1: Figure S4), *OsEV* in rice protoplasts treated by wild type Cas9 with the 20nt esgRNA (Additional file 1: Figure S4) or control rice protoplasts (Fig. 2c ; Additional file 1: Figure S4) |
| OsOD-F  OsOD-R | GGGAGATGAGAGAGCTTGTGCC  GAGTAGTGTAGTACTGAAGAAGCACAGC | Amplifying the *OsOD* target site and 1^st^ PCR for deep sequencing |
| OsOD-A-F  OsOD-A-R | CGATGTCCTTGCTTTCATTCTTCAGTGC  TGACCAGCAGACCAAGATCCCAAGAAC | 2^nd^ PCR for deep sequencing of *OsOD* in rice protoplasts treated by PABE-7 with the native sgRNA (Fig. 1e and Fig. 2a), 14nt esgRNA (Fig. 2c), *OsEV* in rice protoplasts treated by wild type Cas9 with the 14nt esgRNA (Additional file 1: Figure S4) or control rice protoplasts (Fig. 2c ; Additional file 1: Figure S4) |
| OsOD-B-F  OsOD-B-R | ACAGTGCCTTGCTTTCATTCTTCAGTGC  GCCAATGCAGACCAAGATCCCAAGAAC | 2^nd^ PCR for deep sequencing of *OsOD* in rice protoplasts treated by PABE-2 with sgRNA (Fig. 1e), PABE-7 with the esgRNA (Fig. 2a), 15nt esgRNA (Fig. 2c), *OsEV* in rice protoplasts treated by wild type Cas9 with the 15nt esgRNA (Additional file 1: Figure S4) or control rice protoplasts (Fig. 2c ; Additional file 1: Figure S4) |
| OsOD-C-F  OsOD-C-R | CAGATCCCTTGCTTTCATTCTTCAGTGC  CTTGTAGCAGACCAAGATCCCAAGAAC | 2^nd^ PCR for deep sequencing of *OsOD*  in control rice protoplasts (Fig. 1e), or rice protoplasts treated by PABE-7 with the tRNA-sgRNA (Fig. 2a), 16nt esgRNA (Fig. 2c), *OsEV* in rice protoplasts treated by wild type Cas9 with the 16nt esgRNA (Additional file 1: Figure S4) or control rice protoplasts (Fig. 2c ; Additional file 1: Figure S4) |
| OsOD-D-F  OsOD-D-R | AGTCAACCTTGCTTTCATTCTTCAGTGC  AGTTCCGCAGACCAAGATCCCAAGAAC | 2^nd^ PCR for deep sequencing of *OsOD* in rice protoplasts treated by wild type Cas9 with the native sgRNA (Fig. 2c), PABE-7 with the 17nt esgRNA (Fig. 2c), *OsEV* in rice protoplasts treated by wild type Cas9 with the 17nt esgRNA (Additional file 1: Figure S4) or control rice protoplasts (Fig. 2c ; Additional file 1: Figure S4) |
| OsOD-E-F  OsOD-E-R | GTAGAGCCTTGCTTTCATTCTTCAGTGC  GTCCGCGCAGACCAAGATCCCAAGAAC | 2^nd^ PCR for deep sequencing of *OsOD* in rice protoplasts treated by wild type Cas9 with the esgRNA (Fig. 2c), PABE-7 with the 18nt esgRNA (Fig. 2c), *OsEV* in rice protoplasts treated by wild type Cas9 with the 18nt esgRNA (Additional file 1: Figure S4) or control rice protoplasts (Fig. 2c ; Additional file 1: Figure S4) |
| OsOD-F-F  OsOD-F-R | GTTTCGCCTTGCTTTCATTCTTCAGTGC  CGTACGGCAGACCAAGATCCCAAGAAC | 2^nd^ PCR for deep sequencing of *OsOD* in rice protoplasts treated by wild type Cas9 with the tRNA-sgRNA (Additional file 1: Figure S4), PABE-7 with the 19nt esgRNA (Fig. 2c), *OsEV* in rice protoplasts treated by wild type Cas9 with the 19nt esgRNA (Additional file 1: Figure S4) or control rice protoplasts (Fig. 2c ; Additional file 1: Figure S4) |
| OsOD-G-F  OsOD-G-R | ACTGATCCTTGCTTTCATTCTTCAGTGC  ATGAGCGCAGACCAAGATCCCAAGAAC | 2^nd^ PCR for deep sequencing of *OsOD* in control rice protoplasts (Fig. 2a ; Additional file 1: Figure S4) or PABE-7 with the 20nt esgRNA (Fig. 2c), *OsEV* in rice protoplasts treated by wild type Cas9 with the 20nt esgRNA (Additional file 1: Figure S4) or control rice protoplasts (Fig. 2c ; Additional file 1: Figure S4) |
| OsOD-H-F  OsOD-H-R | CAACTACCTTGCTTTCATTCTTCAGTGC  CACCGGGCAGACCAAGATCCCAAGAAC | 2^nd^ PCR for deep sequencing of *OsOD* in rice protoplasts treated by wild type Cas9 with the 14nt esgRNA (Additional file 1: Figure S4), *OsEV* in rice protoplasts treated by wild type Cas9 with the 14nt esgRNA (Additional file 1: Figure S4) or control rice protoplasts (Fig. 2c ; Additional file 1: Figure S4) |
| OsOD-I-F  OsOD-I-R | CAGGCGCCTTGCTTTCATTCTTCAGTGC  CATGGCGCAGACCAAGATCCCAAGAAC | 2^nd^ PCR for deep sequencing of *OsOD* in rice protoplasts treated by wild type Cas9 with the 15nt esgRNA (Additional file 1: Figure S4), *OsEV* in rice protoplasts treated by wild type Cas9 with the 15nt esgRNA (Additional file 1: Figure S4) or control rice protoplasts (Fig. 2c ; Additional file 1: Figure S4) |
| OsOD-J-F  OsOD-J-R | CGGAATCCTTGCTTTCATTCTTCAGTGC  CTAGCTGCAGACCAAGATCCCAAGAAC | 2^nd^ PCR for deep sequencing of *OsOD* in rice protoplasts treated by wild type Cas9 with the 16nt esgRNA (Additional file 1: Figure S4), *OsEV* in rice protoplasts treated by wild type Cas9 with the 16nt esgRNA (Additional file 1: Figure S4) or control rice protoplasts (Fig. 2c ; Additional file 1: Figure S4) |
| OsOD-K-F  OsOD-K-R | GCGCTACCTTGCTTTCATTCTTCAGTGC  TAATCGGCAGACCAAGATCCCAAGAAC | 2^nd^ PCR for deep sequencing of *OsOD* in rice protoplasts treated by wild type Cas9 with the 17nt esgRNA (Additional file 1: Figure S4), *OsEV* in rice protoplasts treated by wild type Cas9 with the 17nt esgRNA (Additional file 1: Figure S4) or control rice protoplasts (Fig. 2c ; Additional file 1: Figure S4) |
| TaDEP1-F  TaDEP1-R | GAGGTCCTACATAATATTTACAGGACCAATTCTCTGG  GGTACGACTTACAGACTAATCCATACATATGGC | Amplifying the *TaDEP1* target site and 1^st^ PCR for deep sequencing |
| TaDEP1-A1-F  TaDEP1-A1-R | TTGCTGGTACTGTACAGAAGAAAGC  CCCACGAAACGTCACTCCAAAATA | Amplifying the *TaDEP1-A1* target site |
| TaDEP1-B1-F  TaDEP1-B1-R | CTTCTTCTTTATGCCTTTGTGCTAGCAGTC  CAACTTCAAGAGCAATATATCGTTTAAG | Amplifying the *TaDEP1-B1* target site |
| TaDEP1-D1-F  TaDEP1-D1-R | AGACAACCTTAATTTGGTCAGTCAGA  ATGCAAGTGGGGTTTGCTGGG | Amplifying the *TaDEP1-D1* target site |
| TaDEP1-A-F  TaDEP1-A-R | CGATGTACCTACAGTGGGTGACTTTTGACAT  TGACCAGTACGACTTACAGACTAATCCATACATATGGC | 2^nd^ PCR for deep sequencing of *TaDEP1* in wheat protoplasts treated by PABE-7 with the native sgRNA (Fig. 2a) |
| TaDEP1-B-F  TaDEP1-B-R | ACAGTGACCTACAGTGGGTGACTTTTGACAT  GCCAATGTACGACTTACAGACTAATCCATACATATGGC | 2^nd^ PCR for deep sequencing of *TaDEP1* in wheat protoplasts treated by PABE-7 with the esgRNA (Fig. 2a) |
| TaDEP1-C-F  TaDEP1-C-R | CAGATCACCTACAGTGGGTGACTTTTGACAT  CTTGTAGTACGACTTACAGACTAATCCATACATATGGC | 2^nd^ PCR for deep sequencing of *TaDEP1* in wheat protoplasts treated by PABE-7 with the tRNA-sgRNA (Fig. 2a) |
| TaDEP1-D-F  TaDEP1-D-R | ATCACGACCTACAGTGGGTGACTTTTGACAT  TTAGGCGTACGACTTACAGACTAATCCATACATATGGC | 2^nd^ PCR for deep sequencing of *TaDEP1* in wheat protoplasts treated by wild type Cas9 with the native sgRNA (Fig. 2c) |
| TaDEP1-E-F  TaDEP1-E-R | ACTTGAACCTACAGTGGGTGACTTTTGACAT  GATCAGGTACGACTTACAGACTAATCCATACATATGGC | 2^nd^ PCR for deep sequencing of *TaDEP1* in wheat protoplasts treated by wild type Cas9 with the esgRNA (Fig. 2c) |
| TaDEP1-F-F  TaDEP1-F-R | TAGCTTACCTACAGTGGGTGACTTTTGACAT  GGCTACGTACGACTTACAGACTAATCCATACATATGGC | 2^nd^ PCR for deep sequencing of *TaDEP1* in wheat protoplasts treated by wild type Cas9 with the tRNA-sgRNA (Fig. 2c) |
| TaDEP1-G-F  TaDEP1-G-R | AGTCAAACCTACAGTGGGTGACTTTTGACAT  AGTTCCGTACGACTTACAGACTAATCCATACATATGGC | 2^nd^ PCR for deep sequencing of *TaDEP1* in control wheat protoplasts (Fig. 2a and Fig. 2c) |
| TaEPSPS-F  TaEPSPS-R | GGGAACAACGGTGGTGGATAACCTGTTG  TATTGACTGCTAATGGAACCAGAGAGCTTAACC | Amplifying the *TaEPSPS* target site and 1^st^ PCR for deep sequencing |
| TaEPSPS-A-F  TaEPSPS-A-R | CGATGTCTCCGTGGAAGCAGATAAAGTTGC  TGACCAATACGTTGCATTTCCACCAGCAG | 2^nd^ PCR for deep sequencing of *TaEPSPS* in wheat protoplasts treated by PABE-7 with the native sgRNA (Fig. 2a) |
| TaEPSPS-B-F  TaEPSPS-B-R | ACAGTGCTCCGTGGAAGCAGATAAAGTTGC  GCCAATATACGTTGCATTTCCACCAGCAG | 2^nd^ PCR for deep sequencing of *TaEPSPS* in wheat protoplasts treated by PABE-7 with the esgRNA (Fig. 2a) |
| TaEPSPS-C-F  TaEPSPS-C-R | CAGATCCTCCGTGGAAGCAGATAAAGTTGC  CTTGTAATACGTTGCATTTCCACCAGCAG | 2^nd^ PCR for deep sequencing of *TaEPSPS* in wheat protoplasts treated by PABE-7 with the tRNA-sgRNA (Fig. 2a) |
| TaEPSPS-D-F  TaEPSPS-D-R | ATCACGCTCCGTGGAAGCAGATAAAGTTGC  TTAGGCATACGTTGCATTTCCACCAGCAG | 2^nd^ PCR for deep sequencing of *TaEPSPS* in wheat protoplasts treated by wild type Cas9 with the native sgRNA (Fig. 2c) |
| TaEPSPS-E-F  TaEPSPS-E-R | ACTTGACTCCGTGGAAGCAGATAAAGTTGC  GATCAGATACGTTGCATTTCCACCAGCAG | 2^nd^ PCR for deep sequencing of *TaEPSPS* in wheat protoplasts treated by wild type Cas9 with the esgRNA (Fig. 2c) |
| TaEPSPS-F-F  TaEPSPS-F-R | TAGCTTCTCCGTGGAAGCAGATAAAGTTGC  GGCTACATACGTTGCATTTCCACCAGCAG | 2^nd^ PCR for deep sequencing of *TaEPSPS* in wheat protoplasts treated by wild type Cas9 with the tRNA-sgRNA(Fig. 2c) |
| TaEPSPS-G-F  TaEPSPS-G-R | AGTCAACTCCGTGGAAGCAGATAAAGTTGC  AGTTCCATACGTTGCATTTCCACCAGCAG | 2^nd^ PCR for deep sequencing of *TaEPSPS* in control wheat protoplasts (Fig. 2a and Fig. 2c) |
| TaGW2-F  TaGW2-R | ATGCCAACCCTTGCGTGTGCGT  TCCTGCTTGTGGGAGCTTTATG | Amplifying the *TaGW2* target site and 1^st^ PCR for deep sequencing |
| TaGW2-A1-F | CTGCCATTACTTTGTATTTTGGTAATA | Amplifying the *TaGW2-A1* target site |
| TaGW2-B1-F | GTTCAGATGGCAATCTAAAAGTT | Amplifying the *TaGW2-B1* target site |
| TaGW2-D1-F | GCATGTACTTTGATTGTTTGCGTGA | Amplifying the *TaGW2-D1* target site |
| TaGW2-A1/B1/D1-R | TCCTTCCTCTCTTACCACTTCCC | Amplifying the *TaGW2* target site |
| TaGW2-A-F  TaGW2-A-R | CGATGTGCAGGAGCAAGGAAGTATAGGAAATC  TGACCAGCGGAACATGTCAAAGACTGGG | 2^nd^ PCR for deep sequencing of *TaGW2* in wheat protoplasts treated by PABE-7 with the native sgRNA (Fig. 2a) |
| TaGW2-B-F  TaGW2-B-R | ACAGTGGCAGGAGCAAGGAAGTATAGGAAATC  GCCAATGCGGAACATGTCAAAGACTGGG | 2^nd^ PCR for deep sequencing of *TaGW2* in wheat protoplasts treated by PABE-7 with the esgRNA (Fig. 2a) |
| TaGW2-C-F  TaGW2-C-R | CAGATCGCAGGAGCAAGGAAGTATAGGAAATC  CTTGTAGCGGAACATGTCAAAGACTGGG | 2^nd^ PCR for deep sequencing of *TaGW2* in wheat protoplasts treated by PABE-7 with the tRNA-sgRNA (Fig. 2a) |
| TaGW2-D-F  TaGW2-D-R | ATCACGGCAGGAGCAAGGAAGTATAGGAAATC  TTAGGCGCGGAACATGTCAAAGACTGGG | 2^nd^ PCR for deep sequencing of *TaGW2* in wheat protoplasts treated by wild type Cas9 with the native sgRNA (Fig. 2c) |
| TaGW2-E-F  TaGW2-E-R | ACTTGAGCAGGAGCAAGGAAGTATAGGAAATC  GATCAGGCGGAACATGTCAAAGACTGGG | 2^nd^ PCR for deep sequencing of *TaGW2* in wheat protoplasts treated by wild type Cas9 with the esgRNA (Fig. 2c) |
| TaGW2-F-F  TaGW2-F-R | TAGCTTGCAGGAGCAAGGAAGTATAGGAAATC  GGCTACGCGGAACATGTCAAAGACTGGG | 2^nd^ PCR for deep sequencing of *TaGW2* in wheat protoplasts treated by wild type Cas9 with the tRNA-sgRNA (Fig. 2c) |
| TaGW2-G-F  TaGW2-G-R | AGTCAAGCAGGAGCAAGGAAGTATAGGAAATC  AGTTCCGCGGAACATGTCAAAGACTGGG | 2^nd^ PCR for deep sequencing of *TaGW2* in control wheat protoplasts (Fig. 2a and Fig. 2c) |
| OsACCT1-OT1-F  OsACCT1-OT1-R | CACTGTCAGTTTATGGTTCCTCAG  CCAGAATCAATCATGTCCTTGACCT | Detecting potential off target effect of site 1 for *OsACC*-T1 |
| OsACCT1-OT2-F  OsACCT1-OT2-R | CAGTGCATTGAAGAGAGATGTGCAT  GGGAGTTCGCTGCAGACCTGA | Detecting potential off target effect of site 2 for *OsACC*-T1 |
| OsACCT1-OT3-F  OsACCT1-OT3-R | CGAGAGAGTACAGCAGCAGC  TCCTCGTGCCACCCCCT | Detecting potential off target effect of site 3 for *OsACC*-T1 |
| F1  R1 | ATGCTCACCCTGTTGTTTGGTGTTACTTC  CTTCTGGGCCTTAATCTCCTGCCTTCT | Detecting PABE-7 construct |
| F2  R2 | TCCGCTACACCAGAGTCTTCTGGAGGATCTAG  GCAGATGGTAGATCGTAGGGTACTTCTCGTGG | Detecting PABE-7 construct |
| F3  R3 | GAAGAACTACTGGCGCCAGCTCCTGAATG  GGCGATCATCTTCCTCACATCGTAAACC | Detecting PABE-7 construct |
| F4  R4 | TCGACAGCCCCACTGTGGCCTACTC  TTATATGCTCAACACATGAGCGAAACCC | Detecting PABE-7 construct |
| F5  R5 | GACCAAGCCCGTTATTCTGAC  TGACCATGATTACGCCAAGCTTAGAC | Detecting pTaU6-esgRNA construct |
| F6  R6 | TGAAAAAGTGGCACCGAGTCGGTGC  GGCGCAGCGGTCGGGCTG | Detecting pTaU6-esgRNA construct |
